# Millisecond Hydrogen/Deuterium-Exchange Mass Spectrometry Approach to Correlate Local Structure and Aggregation in α-Synuclein

**DOI:** 10.1021/acs.analchem.2c03183

**Published:** 2022-11-22

**Authors:** Neeleema Seetaloo, Maria Zacharopoulou, Amberley D. Stephens, Gabriele S. Kaminski Schierle, Jonathan J. Phillips

**Affiliations:** † Living Systems Institute, University of Exeter, Stocker Road, ExeterEX4 4QD, U.K.; ‡ Department of Chemical Engineering and Biotechnology, University of Cambridge, Philippa Fawcett Drive, CambridgeCB3 0AS, U.K.; § Alan Turing Institute, British Library, LondonNW1 2DB, U.K.

## Abstract

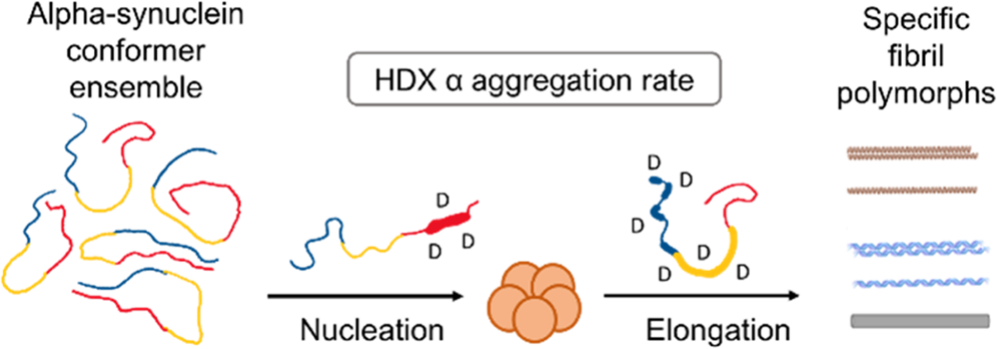

In Parkinson’s disease and other synucleinopathies, α-synuclein misfolds and aggregates. Its intrinsically disordered nature, however, causes it to adopt several meta-stable conformations stabilized by internal hydrogen bonding. Because they interconvert on short timescales, monomeric conformations of disordered proteins are difficult to characterize using common structural techniques. Few techniques can measure the conformations of monomeric α-synuclein, including millisecond hydrogen/deuterium-exchange mass spectrometry (HDX-MS). Here, we demonstrate a new approach correlating millisecond HDX-MS data with aggregation kinetics to determine the localized structural dynamics that underpin the self-assembly process in full-length wild-type monomeric α-synuclein. Our custom instrumentation and software enabled measurement of the amide hydrogen-exchange rates on the millisecond timescale for wild-type α-synuclein monomer up to residue resolution and under physiological conditions, mimicking those in the extracellular, intracellular, and lysosomal cellular compartments. We applied an empirical correction to normalize measured hydrogen-exchange rates and thus allow comparison between drastically different solution conditions. We characterized the aggregation kinetics and morphology of the resulting fibrils and correlate these with structural changes in the monomer. Applying a correlative approach to connect molecular conformation to aggregation in α-synuclein for the first time, we found that the central C-terminal residues of α-synuclein are driving its nucleation and thus its aggregation. We provide a new approach to link the local structural dynamics of intrinsically disordered proteins to functional attributes, which we evidence with new details on our current understanding of the relationship between the local chemical environment and conformational ensemble bias of monomeric α-synuclein.

## Introduction

Parkinson’s disease (PD) is a neurodegenerative condition affecting over 6.2 million people worldwide and this number is predicted to reach 13 million by 2040.^1^ One of the hallmarks of PD is the appearance of cytoplasmic inclusions in neurons, known as Lewy bodies and Lewy neurites, which are mostly constituted of β-sheet-rich aggregates of the protein α-synuclein (aSyn).^2^ In PD and other synucleinopathies, soluble disordered monomeric aSyn can misfold and aggregate, first forming oligomeric species before culminating to insoluble, highly structured amyloid fibrils.^3^ aSyn is a 14.46 kDa protein consisting of 140 amino acid residues divided into three domains: a highly positively charged amphipathic N-terminus (1–60), a central hydrophobic core (61–95) known as the nonamyloid β component (NAC), and an acidic C-terminal tail (96–140) (Figure S1). Unlike well-folded proteins, being natively unfolded, aSyn adopts a broad but shallow conformational space, meaning it can interchange with other conformers with minimal activation energy.^4^ Using a variety of techniques including nuclear magnetic resonance and mass spectrometry, it has been found that the conformations adopted by monomeric aSyn are stabilized by long-range intramolecular electrostatic and hydrophobic interactions between its charged N- and C-termini, and between the C-terminus and the NAC region.^5,6^ Disruptions in these long-range interactions, such as mutations, changes in the local environments and post-translational modifications (PTMs), can skew the conformational ensemble and disturb the stability of the protein, inducing misfolding and aggregation. Therefore, it remains crucial to establish the correlation between monomeric conformation and aggregation propensity/kinetics of aSyn.

While it has been found to be widely distributed in the body,^7^ aSyn is particularly enriched at the presynapse (ca. 20–40 μM)^8^ and has been proposed to participate in the homeostasis and recycling of synaptic vesicles.^9^ aSyn encounters a number of different chemical environments through various routes (summarized in Table 1(10)): (i) exposure to the extracellular space via exocytosis, apoptosis, exosome release, and release of cellular contents;^11^ (ii) endocytosis into the endosomal/lysosomal pathway;^12^ (iii) metabolic imbalances leading to calcium and mitochondrial dysfunction.^13,14^ aSyn in these different environments will have a uniquely biased conformational ensemble.^15^ This leads to the crucial question of whether these different conformational ensembles in the monomer correlate with the propensity and kinetics of aggregation. Furthermore, these differences in structural dynamics of the monomer may result in different fibril morphologies, which could be indicative of alternative aggregation mechanisms.

**Table 1:** Composition of Extracellular, Intracellular, and Lysosomal Compartments Used in This Study (Adapted from Stephens et al.^10^)^a^

		concentration (mM)	
ion	extracellular	intracellular	lysosomal
Na^+^	143	15	20
K^+^	4	140	60
Ca^2+^	2.5	100 nM	0
Mg^2+^	0.7	10	0
pH	7.4	7.2	4.9
buffer	20 mM Tris	20 mM Tris	20 mM citrate

a The maximum potential concentration was used for all ions.

Due to its rapidly interconverting conformations, the structural dynamics of monomeric aSyn is intractable by most structural biology techniques. Hydrogen/deuterium-exchange mass spectrometry (HDX-MS) is one of the few techniques capable of capturing such conformational information on aSyn at high structural resolution.^16,17^ Previous studies on aSyn using HDX-MS have shown that it exchanges on the millisecond timescale and as such requires specialized instrumentation.^18,19^ Here, we obtained data at high structural and temporal resolution for the aSyn monomer under physiologically relevant solution conditions. This was achieved by HDX-MS on the millisecond timescale coupled with a gas-phase “soft fragmentation” technique, electron-transfer dissociation (ETD). We correlated these data with Thioflavin-T (ThT)-based aggregation kinetics and fibril morphology, assessed by atomic force microscopy (AFM). Our results show that the solution conditions assessed in this study all lead to distinct monomeric conformations, aggregation kinetics, and fibril morphologies. More importantly, our correlative analyses reveal specific local conformational changes in the aSyn monomer that influence the separate stages of aggregation, namely, the nucleation and elongation steps.

## Experimental Section

### Materials

All media and reagents were purchased from Sigma-Aldrich (U.K.) and were of analytical grade unless otherwise stated. Deuterium oxide (99.9% D_2_O) was purchased from Goss Scientific (catalogue number: DLM-4). Peptide P1 was synthesized using the method described in Phillips et al.^20^ Details about the expression and purification of wild-type α-synuclein have been described previously.^21^ aSyn refers to the wild-type variant of the protein in this paper. Three biological replicates were produced for use in all experiments.

### Hydrogen/Deuterium-Exchange Mass Spectrometry of α-Synuclein Samples

For labeling times ranging between 50 ms and 5 min, hydrogen/deuterium exchange (HDX) was performed using a fully automated, millisecond HDX labeling and online quench-flow instrument, ms2min^22^ (Applied Photophysics, U.K.), connected to an HDX manager (Waters). For each cellular condition and three biological replicates, aSyn samples in the appropriate equilibrium buffer were delivered into the labeling mixer and diluted 20-fold with labeling buffer at 20°C, initiating HDX. Immediately post-labeling, the labeled sample was mixed with quench buffer in a 1:1 ratio in the quench mixer to arrest HDX. For longer timepoints above 5 min, a CTC PAL sample handling robot (LEAP Technologies) was used. Protein samples were digested onto an Enzymate immobilized pepsin column (Waters) to form peptides. The peptides were trapped on a VanGuard 2.1 mm × 5 mm ACQUITY BEH C18 column (Waters) for 3 min at 125 μL/min and separated on a 1 mm × 100 mm ACQUITY BEH 1.7 μm C18 column (Waters) with a 7 min linear gradient of acetonitrile (5–40%) supplemented with 0.1% formic acid. Peptide samples did not require the initial peptic digestion step. The eluted peptides were analyzed on a Synapt G2-Si mass spectrometer (Waters). An MSonly method with a low collisional activation energy was used for peptide-only HDX. Method parameters were as follows: positive resolution, scan *m*/*z* range 300–2000 Da, scan time 0.3 s, cone voltage 30 V, trap and transfer collision energies fixed at 4 V. An MS/MS ETD fragmentation method was used for HDX-MS-ETD. Method parameters were as follows: ETD fragmentation in positive resolution mode, scan *m*/*z* range 50–2000 Da, scan time 1 s, quadrupole fixed masses (*m*/*z* = 510.9902, 514.0744, 677.8418, 843.2818), transfer collision energy fixed at 8 V. Deuterium incorporation into the peptides and ETD fragments was measured in DynamX 3.0 (Waters).

### ETD Fragmentation of aSyn Peptides

The ETD reagent used was 4-nitrotoluene. The intensity of the ETD reagent per second, determined by the glow discharge settings, was tuned to give a signal of approximately 1e7 counts per second (make-up gas flow: 35 mL/min, discharge current 65 μA) to give efficient ETD fragmentation. Instrument settings were as follows: sampling cone 30 V, trap cell pressure 5e-2 mbar, trap wave height 0.25 V, trap wave velocity 300 m/s, transfer collision energy 8 V, and transfer cell pressure 8e-3 mbar. Hydrogen/deuterium scrambling was measured using Peptide P1 under the same instrument conditions (Figure S2).

### Empirical Adjustment with Bradykinin

The unstructured peptide bradykinin (RPPGFSPFR) was used to calibrate the chemical exchange rate across the four solution conditions as described previously.^23^

### HDX Data Analysis

Raw data was processed, and assignments of isotopic distributions were reviewed in DynamX 3.0 (Waters). The postprocessing analysis was performed using HDfleX. The hybrid significance testing method along with data flattening used here is described elsewhere.^23^

### ThT Binding Assay

ThT kinetic assays were used to monitor the aggregation of aSyn in conditions reflecting the different cellular compartments. All samples were loaded in nonbinding, clear 96-well plates (Greiner Bio-One GmbH, Germany) which were then sealed with a SILVERseal aluminum microplate sealer (Grenier Bio-One GmbH). Fluorescence measurements were taken with FLUOstar Omega microplate reader (BMG LABTECH GmbH, Ortenbery, Germany). Excitation was set at 440 nm, and the ThT fluorescence intensity was measured at 480 nm emission with a 1300 gain setting. The plates were incubated with double orbital shaking for 300 s before the readings (every 60 min) at 300 rpm. Three repeats were performed with six replicates per condition. Each repeat was performed with a different purification batch of aSyn (biological replicate). Data were normalized to the well with the maximum fluorescence intensity for each plate, and the empirical aggregation parameters *t*_lag_, *t*_50_, and *k* were calculated for each condition, based on the equation
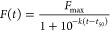
1where *F* is the normalized fluorescence to the highest value recorded in the plate repeat, *F*_max_ is the maximum fluorescence at the plateau, *k* is the slope of the exponential phase of the curve, and *t*_50_ is the time when . One-way ANOVA was used to calculate statistical significance between samples using Prism 8 (GraphPad Software).

### SEC-HPLC

At the end of the ThT-based aggregation assays, the amount of remaining monomer of aSyn in each well was determined by analytical size exclusion chromatography with HPLC (SEC-HPLC). SEC analysis was performed on the Agilent 1260 Infinity HPLC system (Agilent Technologies, U.K.) equipped with an autosampler and a diode-array detector using a AdvanceBio 7.8 mm × 300 mm 130 Å SEC column (Agilent Technologies, U.K.) in 20 mM Tris pH 7.4 at 0.8 mL/min flowrate. Each sample (25 μL) was injected onto the column, and the elution profile was monitored by UV absorption at 220 and 280 nm. The area under the peak in the chromatogram of absorption at 280 nm was determined and used to calculate the monomer concentration. Monomeric aSyn samples spanning from 5 to 40 μM aSyn were used to determine a standard curve, to allow calculation of the protein concentration for the ThT-based aggregation assay samples based on their area under the peak. We also performed SEC-HPLC analysis on the HDX-MS samples (Figure S3). A single peak for monomeric aSyn was observed at 7 min with no peak corresponding to oligomers at ∼5.5 min,^21^ thus confirming that the sample is exclusively monomeric.

### AFM Analysis of Fibril Morphology

Fibrils formed at the end of ThT assays were analyzed by AFM. A freshly cleaved mica surface was coated in 0.1% poly-l-lysine, washed with distilled H_2_O thrice, and dried under a stream of nitrogen gas. Samples from the microplate wells were then incubated for 30 min on the mica surface. The sample was washed thrice in the buffer of choice (for example, in 20 mM Tris, pH 7.4 for the Tris condition) to remove loose fibrils. Images were acquired in fluid using tapping mode on a BioScope Resolve AFM (Bruker) using ScanAsyst-Fluid+ probes. 512 lines were acquired at a scan rate of 1.5 Hz per image with a field of view of 2–5 μm and for at least ten fields of view. Images were adjusted for contrast and exported from NanoScope Analysis 8.2 software (Bruker). Measurements of fibril height and periodicity were performed by cross-sectioning across the fibril and across the fibril axis in NanoScope Analysis 8.2 software (Bruker). Statistical analysis of the height and periodicity measurements was performed in GraphPad Prism 8 (GraphPad Software).

## Results

### Monomer Conformations Vary with Solution Conditions

We hypothesized that the conformational ensemble and local structure of the aSyn monomer could be affecting the aggregation kinetics (as shown in our previous paper^24^) and the resulting fibril morphologies across four conditions with varying pH and ionic compositions, mimicking the extracellular, intracellular, and lysosomal environments, alongside our baseline Tris-only condition (20 mM Tris, pH 7.4). To measure the local (i.e., submolecular) structural and conformational dynamics of the monomer in the different solution conditions, we employed HDX-MS on the millisecond timescale. Protein conformational dynamics influence the exchange of amide hydrogens in the polypeptide backbone, which can be sensitively measured by HDX-MS. The subsecond kinetics are essential to generate data on weakly stable and intrinsically disordered protein monomers, such as aSyn, under physiological conditions—in particular at higher pH found in extracellular and intracellular environments.^25^

We coupled millisecond HDX-MS with “soft fragmentation” by ETD to further increase the structural resolution of the data, with 21% of aSyn resolved at the single amino acid level (Figure S4). Thus, aSyn conformational perturbations can be highly localized to regions of the protein that are involved in specific processes, in this case, aggregation.

Intrinsic amide hydrogen/deuterium-exchange (HDX) varies with pH and ionic strength, which must be corrected to measure only the HDX differences that result from the structural dynamics of the aSyn protein. As described previously,^23^ we used the unstructured peptide bradykinin to empirically calibrate the chemical exchange rate in each solution condition (see SI Methods, Figure S5). Therefore, we were able to robustly determine which significant conformational changes were in monomeric aSyn between the different chemical environments. Briefly, we used the hybrid significance testing method,^26^ combining the results of a Welch’s *t*-test and determining a global significance threshold corresponding to the experimental error, to identify significant differences between the conditions for the deuterium uptake per labeling timepoint and per amino acid (see SI Methods and Seetaloo et al.^23^).

**Figure 1:**
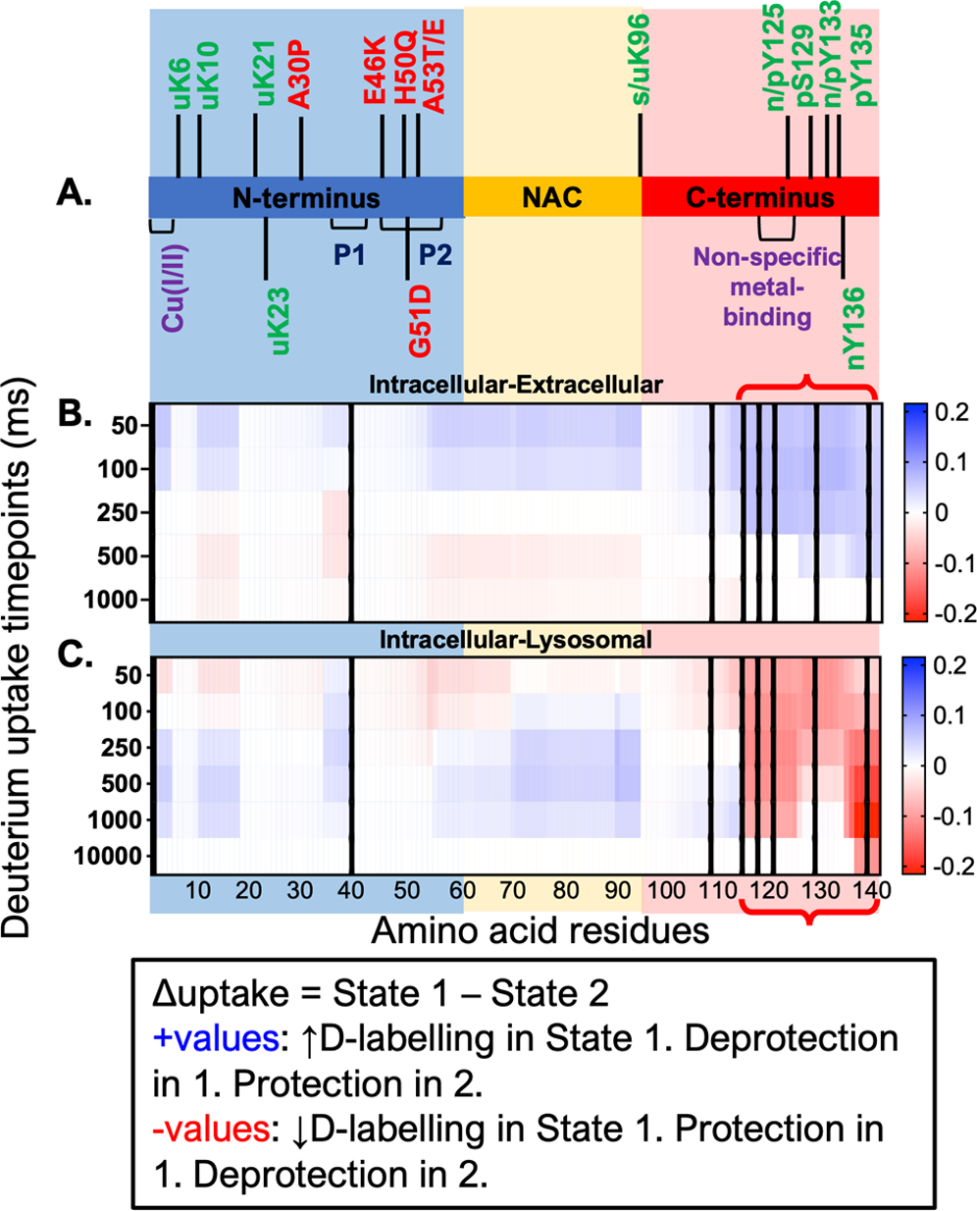
HDX-MS reveals localized differences in conformations of monomeric aSyn across the intracellular, extracellular, and lysosomal conditions. (A) Schematic of aSyn monomer with important features and domains shown. (B, C) Heatmap showing significant differences (nonwhite) in deuterium uptake per timepoint during an on-exchange reaction between STATE 1–STATE 2 (title of each plot). Hybrid significance testing with Welch’s *t*-test *p*-value of 0.05 and global significance threshold of 0.36 Da calculated. Data for three biological replicates shown. Data are resolved to the amino acid level, down to single residues in certain regions. Positive values are in blue and represent decreased uptake in STATE 2, whereas negative values are in red and represent increased uptake in STATE 2. Increased uptake indicates more solvent exposure and/or less participation in stable hydrogen-bonding networks. Tris-only comparisons are shown in Figure S6.

Figure 1 shows the HDX-MS results as a heatmap showing only the significant differences in uptake at each experimental timepoint, from 50 ms to 10 s, in a pairwise manner between the intracellular, extracellular, and lysosomal conditions. Part of the C-terminus is significantly protected in the extracellular state, compared to the intracellular state (blue residues in Figure 1B). Conversely, the N-terminus and NAC residues 2–4, 10–17, 34–38, and 53–94 are deprotected (red residues in Figure 1B). A similar pattern in the opposite direction is seen for the intracellular vs lysosomal differential across residues 1–112, with the remainder of the C-terminal sequence showing a slightly different pattern of uptake difference. On the other hand, the comparisons of Tris-only versus all of the physiological states (Figure S6) show protection against HDX throughout, with highest protection conferred to the C-terminus. The differential HDX-MS analysis confirms that the aSyn monomer varies in conformational ensemble across the physiological and Tris-only conditions studied here and localizes the ensemble-averaged conformational changes.

### Aggregation Propensity Increases from Tris-Only < Extracellular < Intracellular < Lysosomal Conditions In Vitro

**Figure 2:**
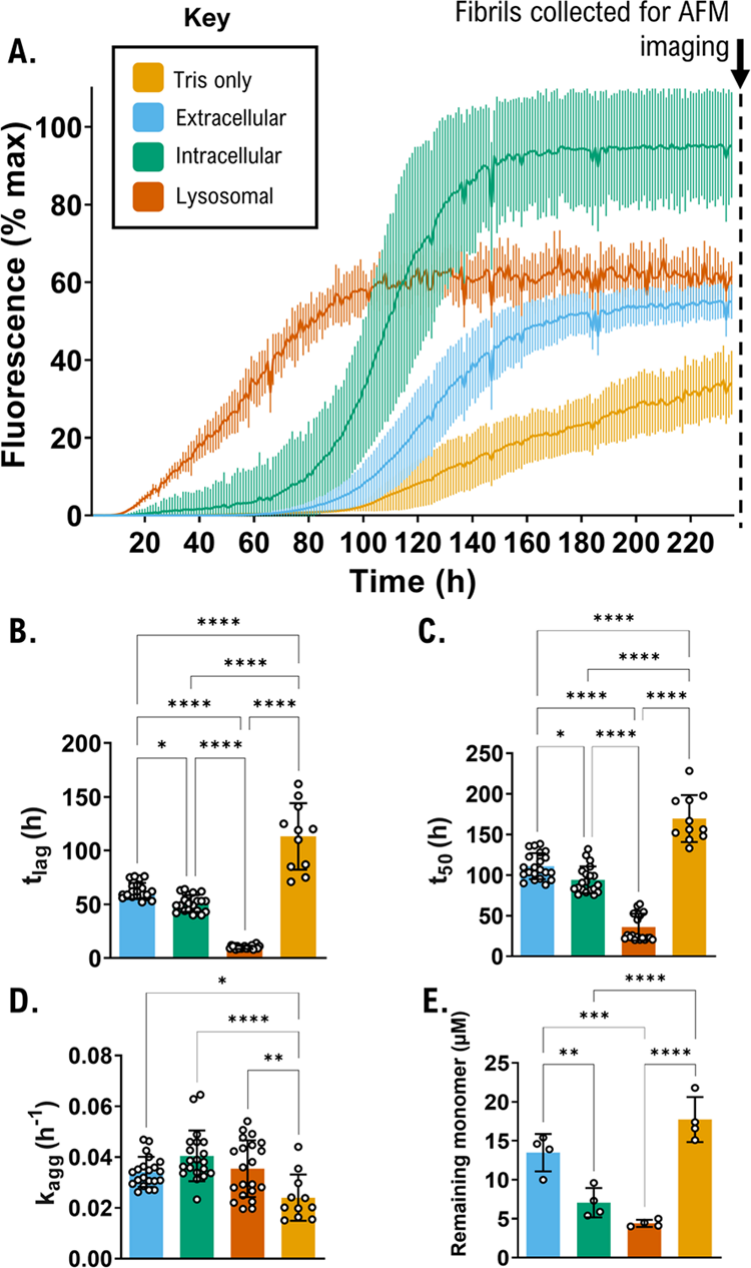
ThT-based aggregation assays reveal distinct aggregation behavior for aSyn when equilibrated in different physiological solution conditions. (A) Aggregation kinetics of aSyn in Tris-only (yellow), extracellular (blue), intracellular (green), and lysosomal (orange) solution conditions were measured using ThT fluorescence intensity and plotted as % of maximum fluorescence at 480 nm. Trace shows average and standard deviation of up to nine technical replicates. Biological replicate 1 shown (see Figure S8 for all biological replicates); (B–E) Lag time (*t*_lag_), time to reach 50% of maximum aggregation (*t*_50_), and slope (*k*_agg_) were calculated, and significance testing was performed by a one-way ANOVA with Tukey’s multiple comparisons post hoc test. The upper and lower 95% confidence interval is shown and *p*-value significance of differences between cellular conditions are indicated (*p < 0.05, **p < 0.01, ***p < 0.001, ****p < 0.0001). The remaining monomer concentration was determined using SEC-HPLC by injecting 25 μL of soluble sample from each well in the ThT assay and calculating the area of the aSyn monomer peak in relation to a standard curve of known aSyn monomer concentrations. Remaining monomer concentrations were measured from the area under the peak and calculated using a standard curve of known concentrations. Data shown in (B)–(E) correspond to *n* = 21 for the extracellular, intracellular, and lysosomal conditions, and *n* = 11 for Tris-only.

We next investigated whether the aggregation propensity of aSyn differed across the solution conditions. To do so, we used a ThT-based fluorescence assay. The ThT molecule emits fluorescence when bound to rich fibrillar β-sheet structures, informing us on the process of aggregation^27^ (Figure 2). In the ThT-based assay, the time before the onset of fluorescence, lag time (*t*_lag_), is indicative of the nucleation phase of fibril formation and the slope of the exponential growth (*k*_agg_) describes the elongation phase (Figure S7). Upon the addition of physiologically relevant salts, the aSyn monomer nucleation lag time is reduced by 45% from 113 h to 62 h and the elongation rate *k*_agg_ is increased by 42% (0.024–0.034 h^–1^), as can be seen from Figure 2B,D, respectively.

As ThT-based fluorescence intensity can change due to the presence of different fibril polymorphs and solution conditions,^28,29^ we confirmed the extent of aggregation by quantifying the remaining monomer concentration at the end of the assays (Figure 2E). Thus, the aggregation propensity can be described as the reciprocal of the remaining monomer. The order of aggregation propensity from highest to lowest was: Lysosomal > Intracellular > Extracellular > Tris-only. Importantly, the cellular and extracellular compartment conditions all had a higher aggregation propensity than the Tris-only condition, showing that when deprived of biological salts, Tris-only is not physiologically relevant despite being at a physiological pH of 7.4. This may be particularly significant for drug discovery efforts which often use aSyn protein in buffers without a full complement of dissolved physiological salts corresponding to the relevant physiological compartment. We also note that aggregation propensity correlates with pH—the lower the pH, the greater the aggregation propensity.

The lysosomal condition corresponds to the fastest aSyn aggregation rate, as previously shown.^30^ The different aggregation kinetics and propensities that we observed logically provoke the question as to whether they also result in different aSyn fibril polymorphs; thus, we next imaged the fibrils in each case.

### Different Physiological Conditions Result in Five Distinct Fibril Polymorphs

**Figure 3:**
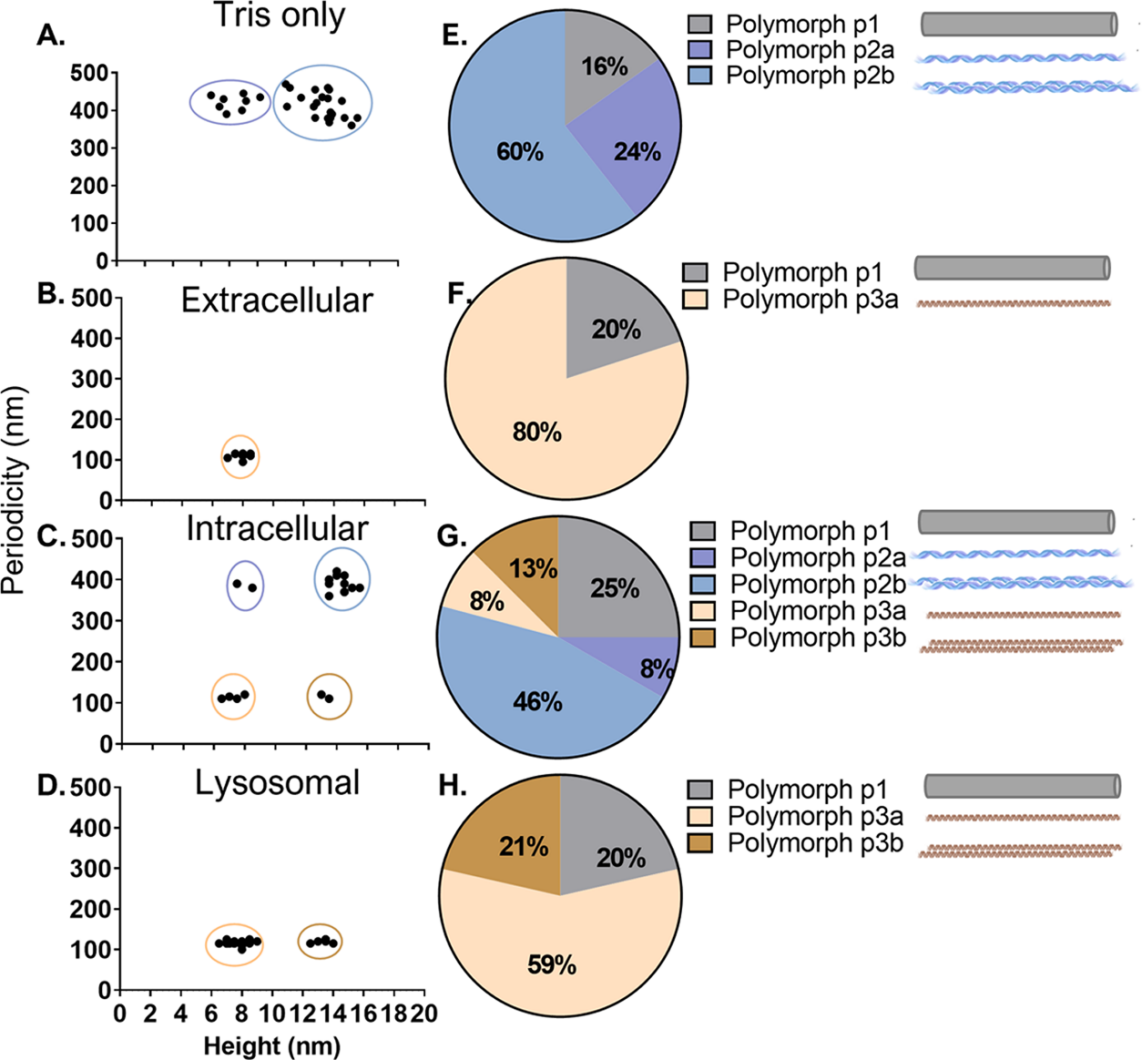
AFM analysis on the aSyn fibrils formed from each condition reveals distinct polymorphs. Atomic force microscopy was performed on the fibrils developed in each cellular, extracellular, and Tris-only condition. (A–D) Plots of the periodicity against the height in nm for each condition. As a guide to the eye, groups of distinct polymorphs are highlighted by a color ellipse. Nonperiodic fibrils (or rods) are not depicted. (E–F) Pie charts representing the abundance of each polymorph population and cartoons of polymorphs associated with each condition. Polymorph p1 (gray) represents fibril rods, while polymorphs p2–p3 are twisted fibrils of varying periodicities and heights, with colors matching the cluster circles in (A–D). AFM images of the main fibril polymorph in each condition can be found in Figure S9.

Next, we examined the fibrils formed under each condition to identify any resulting morphological variations. As previous studies have shown, the morphology of aSyn fibrils is highly sensitive to solution conditions such as pH and ionic composition.^31,32^ The properties of the different polymorphs such as toxicity and seeding potency may differ.^33^ The AFM analysis showed that all conditions had a percentage of the total population as nonperiodic, or rod fibrils, that we termed polymorph p1 (Figure 3). Tris-only fibrils were predominantly composed of twisted polymorphs p2 (Figure 3A,E). These were divided into two subpolymorphs p2a and p2b, as they both had a long periodicity of ∼400 nm, but had different heights, with polymorph p2b (12–17 nm) having approximately double the height of polymorph p2a (7–10 nm). This can be rationalized by two protofibrils (p2a) associating to form a mature fibril (p2b).^34^ Polymorph p2b formed 60% of the total fibril population, with the lower height p2a a further 24% and the rod polymorph p1 making up only 16% (Figure 3E). The extracellular conditions created a single population of protofibrils containing the periodic polymorph p3a, which was more tightly twisted than the Tris-only fibrils, with a short periodicity of ∼100 nm (Figure 3B). None of the protofibrils were found with heights more than 8 nm, suggesting they had not laterally associated or twisted together under extracellular conditions. The lysosomal condition created two periodic fibril populations: (i) polymorph p3a; indistinguishable from the extracellular condition and (ii) polymorph p3b; a mature fibril of the same periodicity but double the height of protofibril p3a (Figure 3D). In both, the extracellular and the lysosomal conditions, most of the fibril populations were of p3a (7–9 nm), comprising 80 and 59%, respectively (Figure 3F,H). The intracellular fibril population was more diverse and included polymorphs found across the three other conditions, with the majority (46%) of the fibrils being p2b (Figure 3C,G).

The AFM analysis shows that the cellular, extracellular, and Tris-only conditions cause aSyn to form fibrils with five distinct morphologies, p1, p2a, p2b, p3a, and p3b.

### Exposure of C-Terminus Residues 115–135 Are Key for Nucleation

We then sought to correlate the localized structural perturbations in the monomeric aSyn with the nucleation and elongation phases of the aggregation kinetics. We aimed to determine if there were certain structural motifs or regions in the aSyn monomer whose protection or deprotection to HDX reveals a contribution to each aggregation phase.

**Figure 4:**
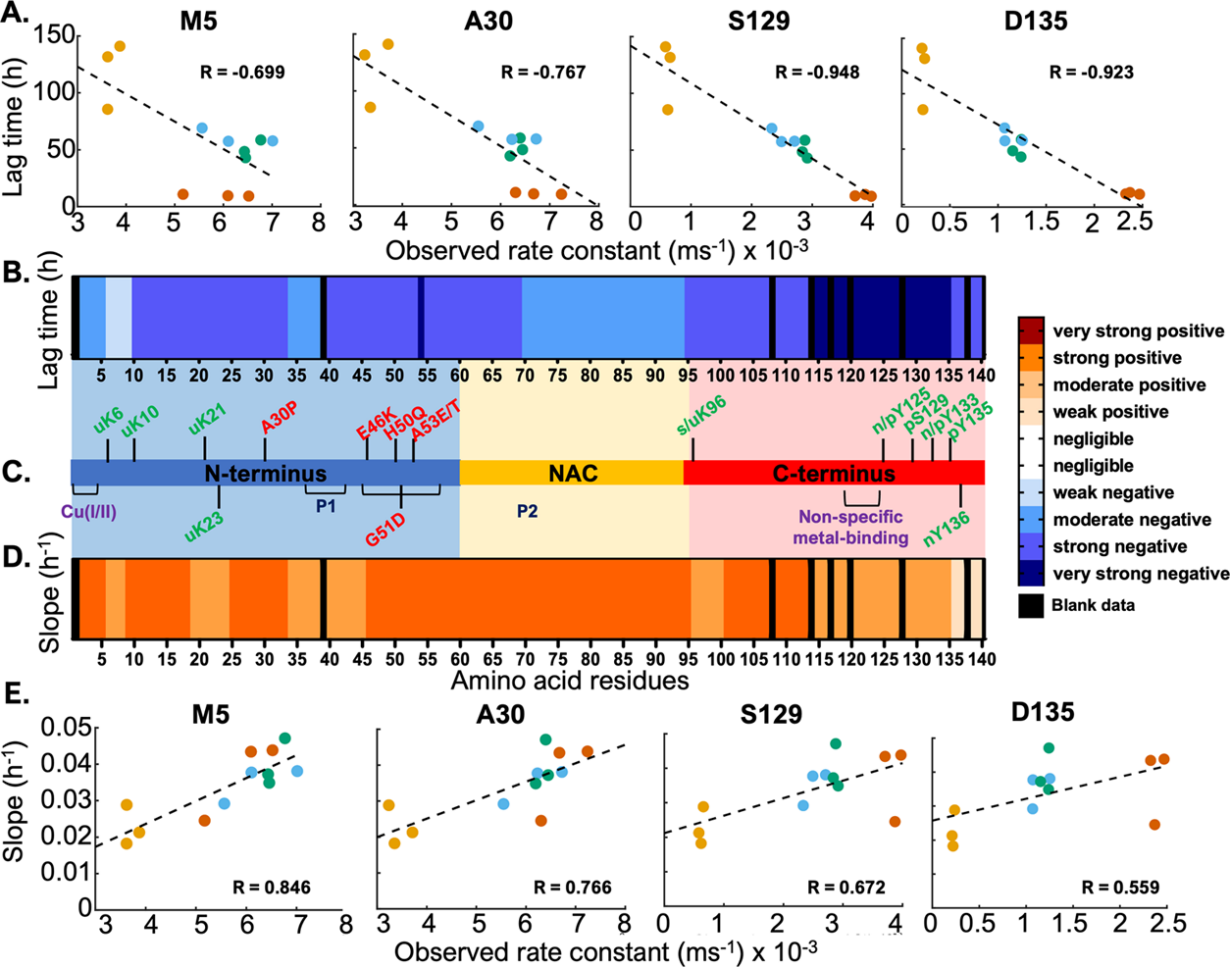
Correlation analysis confirms regions of the protein important to the nucleation and elongation phases of the aggregation kinetics. (A) Correlation plots of lag time against *k*_obs_ for selected amino acid residues—M5 (copper-binding site), A30 (familial mutation site), S129 and D135 (post-translational modification sites); (B) heatmap of the Pearson correlation coefficients (*R*) between the lag time and *k*_obs_; (C) schematic of the aSyn sequence showing the three domains (N-terminus in blue, NAC in yellow, C-terminus in red), sites of selected familial mutations (red), metal-binding (purple) and post-translational modifications (green); (D) heatmap of the Pearson correlation coefficients (*R*) between the aggregation slope and *k*_obs_; (E) correlation plots of aggregation slope against *k*_obs_ for selected amino acid residues (same as A). Color bar legend is shown with the following categories of *R*: negligible: 0–0.3, weak: 0.3–0.5, moderate: 0.5–0.7, strong: 0.7–0.9, very strong: 0.9–1. Black regions represent unavailable data.

We performed a Pearson correlation analysis at each amino acid in aSyn, with a 99% confidence limit, between the nucleation lag time (*t*_lag_) from ThT-based assays (Figure 2) and the observed rate constant (*k*_obs_) of hydrogen-exchange (Figures 4A,B and S10). Table S1 shows the Pearson correlation coefficients *R* at each amino acid. C-terminus residues 115–135 are very strongly negatively correlated with *t*_lag_ (*R* < −0.9), while the rest of the C-terminus is strongly negatively correlated (−0.9 < *R* < −0.7), albeit to a lesser extent (Figure 4B). Similarly, certain localized regions of the N-terminus (10–33 and 40–60) and the NAC region (61–69) are also strongly negatively correlated (−0.9 < *R* < −0.7), but to a lesser extent. Therefore, aSyn conformations, where the above-mentioned residues are exposed and/or their hydrogen-bonding networks are destabilized, are found to nucleate more rapidly.

### Exposure of N-Terminus and NAC Regions Drives Fibril Elongation

We next correlated the rate of fibril growth, defined by the slope of the exponential phase (*k*_agg_) from the ThT-based assays with the observed rate constant (*k*_obs_) as before. We have performed a Pearson correlation analysis, as above, and show a heatmap of the correlation coefficients *R* from the *k*_obs_–*k*_agg_ correlation along the protein sequence (Figure 4D,E). A strong positive correlation (0.7 < *R* < 0.9) can be seen throughout the entire NAC region (residues 61–95) and for N-terminus residues 2–5, 10–17, 25–33, and 46–60. The C-terminal domain residues 101–113 also showed strong positive correlation coefficients (0.7 < *R* < 0.9) compared to the rest of the protein sequence. This means that the more exposed or less involved in hydrogen-bonding these residues, the higher the elongation rate, implying faster fibril growth. Interestingly, C-terminal residues 115–135 that previously proved to be critical for the nucleation phase are only moderately influential for the process of fibril elongation. Thus, monomeric conformations where the NAC region, together with the above-mentioned sites, is exposed to solvent water and/or has a destabilized H-bonding network, are found to accelerate fibril elongation.

## Discussion

We correlated the different cellular aggregation profiles from the ThT-based assays with the *k*_obs_ from HDX-MS and discovered that the C-terminal residues 115–135 crucially influenced the nucleation of the fibrils, as shown by the very strong correlation coefficients spanning this region. Previous studies on aSyn, involving mutated, phosphorylated, and truncated variants, have shown that the truncation or charge neutralization of the C-terminus increases the rate of aggregation by decreasing the lag time and increasing the extent of fibrillation.^35−39^ As we saw, the *k*_obs_ for HDX at the C-terminus in full-length wild-type aSyn strongly negatively correlated with *t*_lag_, indicating that the more exposed it is or the less involved in stable H-bonding network, the faster the nucleation. Upon charge reduction at the C-terminus (possibly via calcium-binding or lowering to lysosomal pH), a drop in the long-range electrostatically stabilized interactions between N- and C-terminus regions may occur. This would lead to increased solvent exposure and/or destabilized H-bonding networks, resulting in faster nucleation. Correspondingly, some residues (2–5, 10–18, 25–33, 46–60, 61–95, and 100–113) were found to promote fibril growth when deprotected against HDX (deep orange in Figure 4D). Perhaps unsurprisingly, the entire NAC was found to be highly important in the process of fibril growth, in agreement with previous deletion and truncation studies.^40^ Furthermore, recent studies have identified two motifs at the N-terminus, P1 (residues 36–42) and P2 (residues 45–57) to be critical for aggregation.^41^ Our high-resolution analysis confirms that exposure of motif P2 drives both nucleation and fibril growth processes of aggregation, and to a higher extent than that of motif P1, in full-length wild-type aSyn. Therefore, our novel correlative analyses on the full-length wild-type aSyn were able to conclude the same as previous truncation, deletion, and mutation studies and localize the structural dynamics of monomeric aSyn to different stages of aggregation, without altering the protein sequence.

Interestingly, we observed that fibrils formed under extracellular and lysosomal conditions led to the same more tightly twisted fibrils, polymorph p3.^42^ The only significant difference between the aggregates formed under these two conditions was the propensity of the lysosomal buffer to drive the assembly of p3a protofibrils into p3b mature fibrils. In common, both conditions lead to a net charge reduction at the C-terminus, either by calcium-binding^43^ or neutralization of certain acidic residues at the lower pH,^44^ respectively, which would disrupt the long-range electrostatic and hydrophobic interactions that stabilize the monomer in solution.^24^ It is likely that a change in the protofibril structure and/or charge halts the formation of mature fibrils by affecting their association. CryoEM studies have revealed the formation of a different aSyn polymorph upon the E46K point mutation, which led the protofibrils to adopt a different fold compared to previously resolved wild-type aSyn structures.^45,46^ It is possible that a different monomer conformation (lysosomal vs extracellular) could lead to different protofibril packing and reduced stability of the mature fibril. This suggests that the same aggregation pathway may be followed to generate the p3a fibrils from the aSyn monomer in the extracellular and lysosomal environments, but that the mature fibrils have considerably higher stability under the lysosomal conditions. From our HDX-MS vs ThT correlative analyses, the C-terminus deprotection was also found to correlate with the nucleation phase of aggregation, agreeing with previous work.^36,47,48^ Therefore, we can infer that polymorph p3 is determined by an aSyn monomeric conformation with a C-terminus with a lower net charge during the nucleation phase. The intracellular condition formed the most heterogeneous fibril populations out of the four conditions, as it had all of the polymorphs of the other conditions combined. It also gives rise to the widest range of fibril elongation rates (Figure 1E). The ensemble average of structural conformers, as measured by HDX-MS, was broadly similar between intra- and extracellular conditions; however, the intracellular environment stabilizes specific sites in the N-terminal region and to a far greater degree destabilizes the C-terminal region (Figure 1B). The C-terminal protection can be attributed to calcium binding.^49^ The intracellular state also contains Mg^2+^, which is known to bind to aSyn.^15^ It is possible that in this case, Ca^2+^ binds preferentially to the Mg^2+^, but this statement can only be confirmed if a direct comparison of the two ions is performed (e.g., Tris + Ca^2+^ vs Tris + Mg^2+^). Together, these results suggest that the intracellular state stabilizes aSyn in a relatively diverse set of monomeric conformations and net charge states and that these aggregate into a heterogeneous mixture of fibrils, which could be associated with different biophysical properties, levels of toxicity, and disease relevance.

It is important to note that while this study presents correlations between local structural dynamics and aggregation in full-length wild-type aSyn, there are a wide variety of familial mutations, post-translational modifications, and even different physiological buffers—all of which have the potential to change those site-specific correlations. For example, in the case of mutation H50Q, where a basic residue is swapped for an amidic one, the electrostatics are changed with the removal of a formal charge, which may impact the specific chemistry involved in nucleation/elongation processes, and the observed rate constant would decrease by 4.2x based on the intrinsic rates documented by Bai et al.^50^ This would likely affect the correlation at this residue and any other structurally connected sites elsewhere in the protein. Thus, each aSyn variant and the chemical environment should be considered nontrivial to extrapolate and each deserves assessment.

In the present study, we described a new approach using millisecond HDX-MS coupled to ETD “soft fragmentation” to correlate local structural conformations with aggregation kinetics for full-length wild-type aSyn. While it may be of significant merit to extend these data to directly address the local structural perturbations of deletions, truncations, and mutations, a particular strength of this approach is the ability to obtain highly resolved structural dynamics information on unmodified wild-type protein.^35−39^ This strategy can provide evidence of the relationship between a functional attribute and local structural features that stem from a bias in the conformer ensemble under physiologically relevant conditions. Thanks to this approach, we found that deprotection in the center of the C-terminal domain was found to be significantly correlated with the nucleation phase of the aggregation kinetics and we identified specific residues that influenced fibril growth in full-length wild-type aSyn. We also discovered that the morphology of certain fibril polymorphs was determined during monomer nucleation. We anticipate that in the future, the tools and generally applicable approach that we present here will be able to make further important structure–function correlations for other physiological conditions and proteoforms of aSyn and intrinsically disordered proteins more widely.

## Supporting Information Available

Additional method details (HDX-MS sample preparation, HDfleX data analysis, ThT assay sample preparation, code for correlation analyses); aSyn charge distribution, H/D scrambling test, schematic of aggregation phases of nucleation and elongation, combined ThT assay results for three biological replicates, AFM images, structural resolution map of aSyn from combined peptide and ETD HDX-MS data, chemical exchange rate calibration with unstructured peptide bradykinin, absolute uptake plots for monomeric aSyn under each condition, HDX differences between Tris-only and cellular compartment conditions, empirically adjusted deuterium uptake plots; Pearson correlation coefficients and code for their calculation, number of fibril polymorphs identified per condition, and HDX-MS experimental technical details (PDF)
ThT, AFM, and HDX mass spectrometry source data (XLSX)

## Notes

The authors declare that the data supporting the findings of this study are available in this paper and its Supporting Information files. Source data are provided with this paper. All mass spectrometry .raw files will be available from the PRIDE repository [accession pending]. This study uses in-house-developed software available to download: [http://hdl.handle.net/10871/127982].^23^
