# Supplementary material for: Millisecond Hydrogen/Deuterium-Exchange Mass Spectrometry Approach to Correlate Local Structure and Aggregation in α-Synuclein
Source: Anal Chem. 2022 Nov 22;94(48):16711–9. doi: 10.1021/acs.analchem.2c03183 (PMC9730295; doi:10.1021/acs.analchem.2c03183)
Supplement: Supplementary file 1 — ac2c03183_si_001.pdf [file ac2c03183_si_001.pdf]

Supporting Information

A millisecond hydrogen/deuterium-exchange mass spectrometry approach to correlate local structure and aggregation in alpha-synuclein

Neeleema Seetaloo[1†], Maria Zacharopoulou[2†], Amberley D. Stephens[2], Gabriele S. Kaminski Schierle[2], Jonathan J. Phillips[1,3]*

[1]Living Systems Institute, University of Exeter, Stocker Road, Exeter, EX4 4QD, UK

[2]Department of Chemical Engineering and Biotechnology, University of Cambridge, Phillipa Fawcett Drive, Cambridge, CB3 0AS, UK

[3]Alan Turing Institute, British Library, London, NW1 2DB, UK

[†]These authors contributed equally.

*Corresponding author: jj.phillips@exeter.ac.uk

Table of Contents

METHODS 3

Sample preparation 3

Data analysis 3

ThT sample preparation 3

Figure S1: Charge distribution over the alpha-synuclein sequence over pH 7.4 (blue), 7.2 (green) and 4.9 (orange). 4

Figure S2: Hydrogen-deuterium scrambling is not observed in the c and z fragments of peptide P1 under identical conditions as aSyn experiments. 5

Figure S3: SEC-HPLC confirms the protein remains primarily monomeric under the HDX-MS conditions. 6

Figure S4: Structural resolution of aSyn HDX data. 7

Figure S5: Unstructured peptide bradykinin calibrates chemical exchange rate effects 8

Figure S6: HDX-MS reveals localised differences in conformations of monomeric aSyn across Tris only vs the compartment conditions. 9

Figure S7: Schematic of the aggregation phases of nucleation (lag time) and elongation (slope of curve). 10

Figure S8: Average of three biological replicates upholds the same trend as summary data in Figure 1. 11

Figure S9: Representative AFM images of the main fibril polymorph in each condition. Note scale bars and height colour bars are not identical. 12

Figure S10: HDX-MS reveals different conformations in monomeric aSyn across all the conditions. 13

Table S1: Pearson correlation coefficients R along aSyn protein sequence (including ETD data) for correlation analyses between observed rate constant kobs from HDX-MS and nucleation lag time (t_{lag}) and elongation rate (k_{agg}) from ThT assays. 14

Figure S11: Empirically adjusted deuterium uptake plots for aSyn equilibrated in Tris only, extracellular, intracellular, and lysosomal buffer conditions. 19
Table S2: Number of fibril polymorphs (n) identified per condition. 20
Table S3: HDX-MS experimental technical details. 20
Code used to calculate the Pearson correlation coefficients 21
References 23

METHODS

Sample preparation

Four buffer conditions were used in this study: Tris-only, extracellular, intracellular and lysosomal[1] (Table 1). aSyn samples from three different purification batches were removed from -80˚C storage (15-25 µM stocks in Tris-only equilibrium buffer). For the Tris-only sample, the protein concentration was adjusted to 5 µM with Tris-only equilibrium buffer. For the extracellular sample, the salts were directly diluted into the 5 µM protein sample. For the intracellular and lysosomal samples, the protein was buffer exchanged into the matching equilibrium buffer for six cycles using 3K MWCO regenerated cellulose Amicon ultra centrifugal filters (Millipore, USA) and made to 5 µM.

Data analysis

In HDfleX, briefly, the back-exchange-corrected data points for each peptide and ETD fragment were fitted using equation 1 in one-phase.

$$D_t = N \sum_{\mathrm{i}=1}^{nExp} \left[1 - e^{-(k_{obs,i}t)^{\beta_i}} \right] \qquad \text{Equation 1}$$

where D_t is the deuterium incorporation at time t, $nExp$ is the number of exponential phases, N is the maximum number of labile hydrogens, k_{obs} is the observed exchange rate constant and β is a stretching factor.

As the rate of HDX is affected by pH and ionic strength, which are not controlled in this study, it is crucial to normalize the solution effects between the different conditions being compared. Here, we used an empirical approach to normalization using the unstructured peptide bradykinin (RPPGFSPFR)[2,3] to deconvolute the solution effects of the HDX from the protein structural changes. Due to the unstructured nature of bradykinin, all the differences in deuterium uptake seen from the different buffers can be assumed to be strictly from the changes in the chemical exchange rate effects, rather than structural effects. By using bradykinin to calibrate the chemical exchange rate, we can now clearly distinguish the structural changes between the cellular conditions (Figure S3). The ETD fragments were combined with the peptide data using HDfleX to give the absolute uptake information across the entire protein as in Figure S4.

ThT sample preparation

40 µM (final concentration) of freshly made ThT solution (Abcam, Cambridge, UK) in distilled water was added to 50 µL of 40 µM, aSyn in 20 mM Tris pH 7.4, extracellular, intracellular, and lysosomal conditions as described in Table 1.

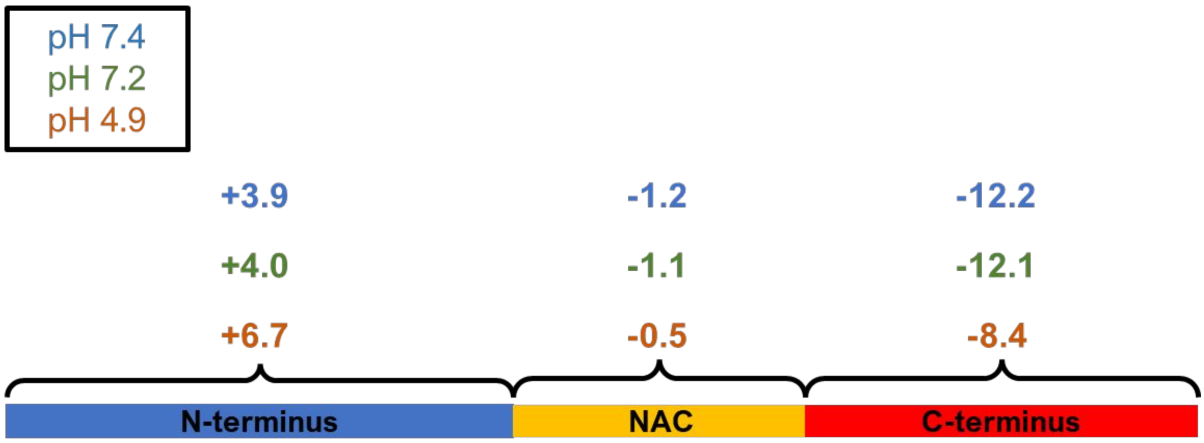

Figure S1: Charge distribution over the alpha-synuclein sequence over pH 7.4 (blue), 7.2 (green) and 4.9 (orange).

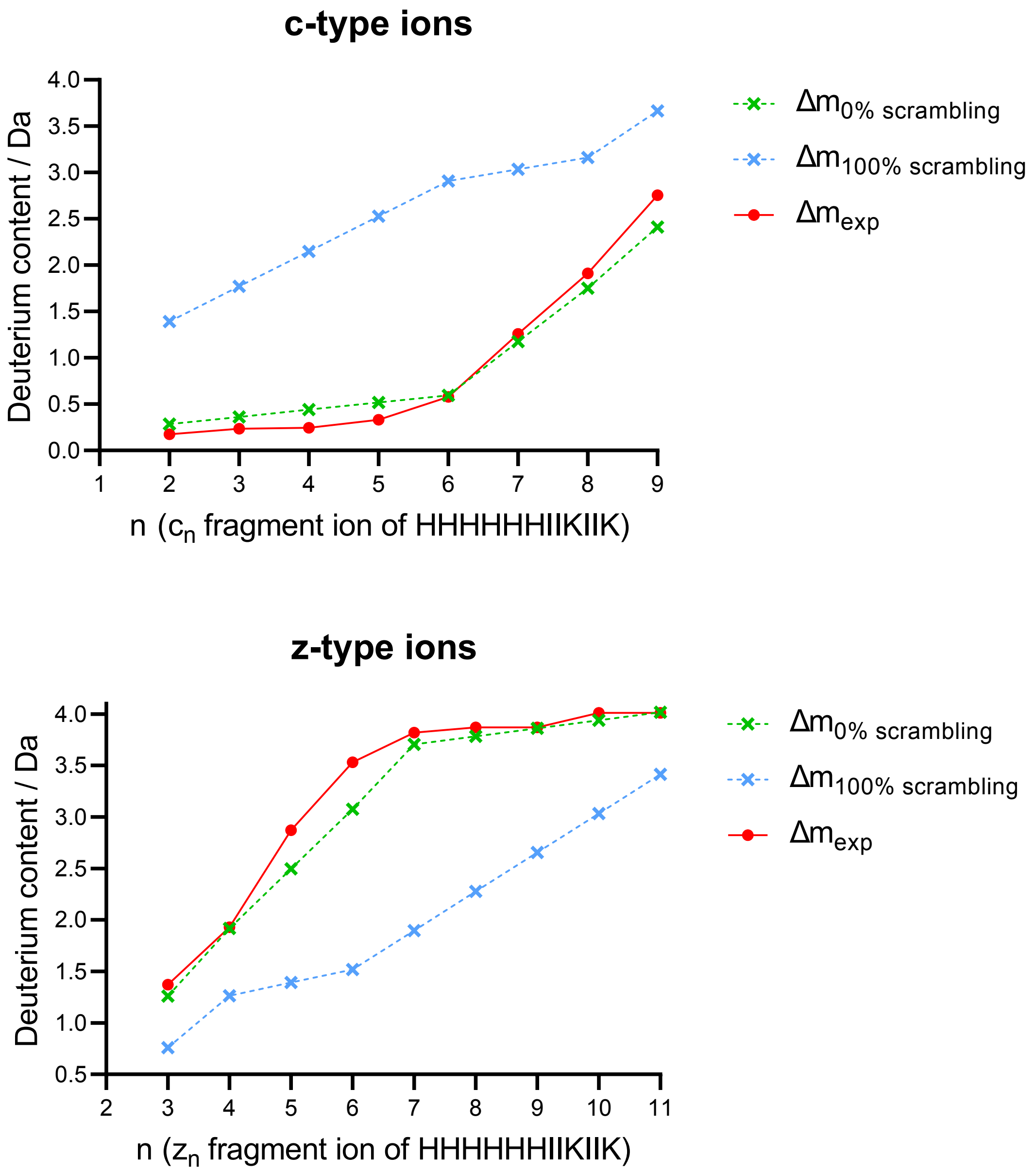

Figure S2: Hydrogen-deuterium scrambling is not observed in the c and z fragments of peptide P1 under identical conditions as aSyn experiments. The green and blue dotted lines on the plots represent the 0% and 100% theoretical scrambling data for this peptide respectively, and the red line shows the experimental data. From the plots, it can be inferred that the conditions chosen for ETD fragmentation do not cause H/D scrambling as the experimental data line (red) approaches the 0% scrambling line (green) for both types of fragments.

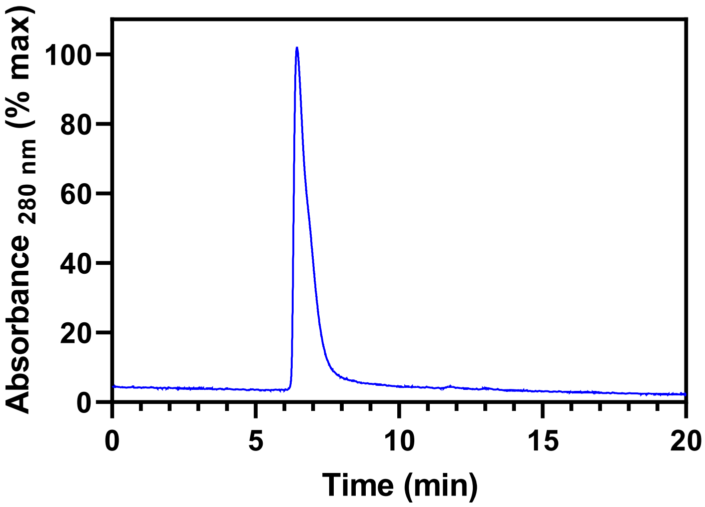

Figure S3: SEC-HPLC confirms the protein remains primarily monomeric under the HDX-MS conditions. 25 µM aSyn was defrosted, filtered at 0.22 µm and allowed to equilibrate at 4˚C for 12 hours. SEC analysis was performed on the Agilent 1260 Infinity HPLC system (Agilent Technologies, UK) equipped with an autosampler and a diode-array detector using an Advance-Bio 7.8 × 300mm 130 Å SEC column (Agilent Technologies, UK) in 20 mM Tris pH 7.4 at 0.8 mL/min flowrate. 25 µL of sample was injected onto the column and the elution profile was monitored by UV absorption at 220 and 280 nm. The area under the peak in the chromatogram of absorption at 280 nm was determined and used to calculate the monomer concentration. A single peak is observed in the chromatogram, confirming that the protein remains primarily monomeric under the conditions used here.

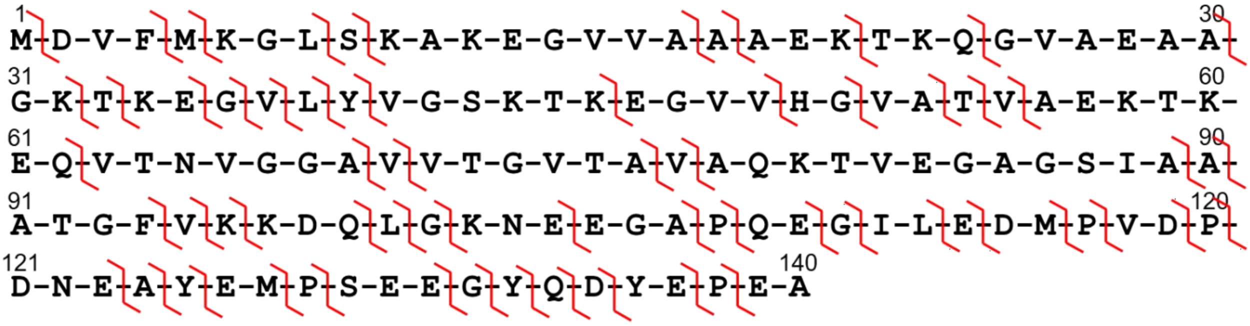

Figure S4: Structural resolution of aSyn HDX data. Red ticks represent the sites of either proteolytic cleavage or ETD fragmentation. The merged resolution obtained by combining peptides and c/z fragments is given by the length of the gaps between these red ticks – shorter gaps indicate higher structural resolution, with 21% of the aSyn protein at single amino acid resolution.

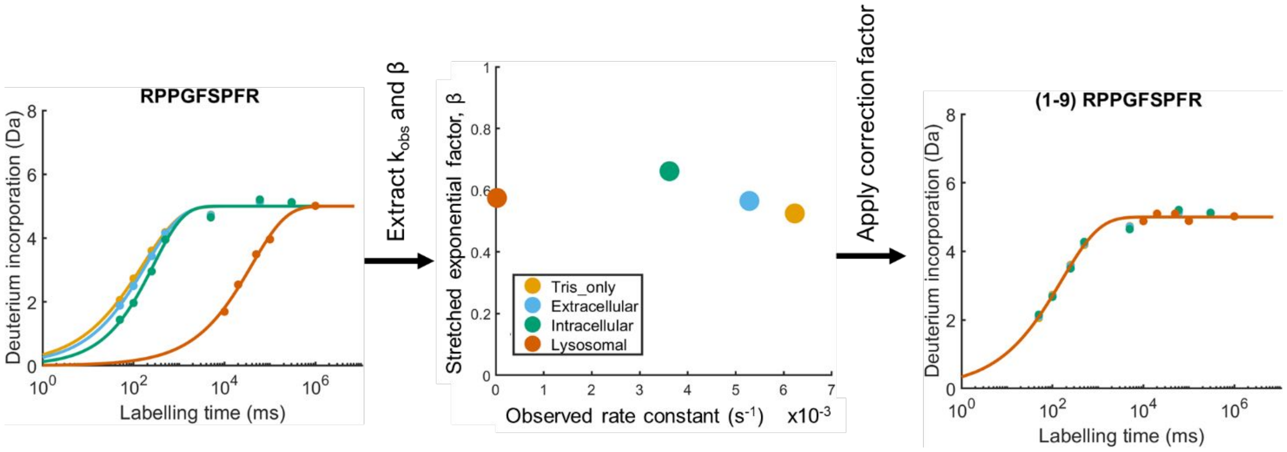

Figure S5: Unstructured peptide bradykinin calibrates chemical exchange rate effects; left plot: uptake curve for bradykinin in the four conditions; middle plot: 2D plot of extracted fitted parameters k_{obs} and β, with Tris (yellow dot) chosen as reference state; right plot: empirical correction applied to all states. Data points are the mean of n=3 technical replicates. Note that in the right plot several data points are not visible due to overlap of all data collected at any given labelling time after the correction for the different intrinsic HDX chemistry under the different conditions.

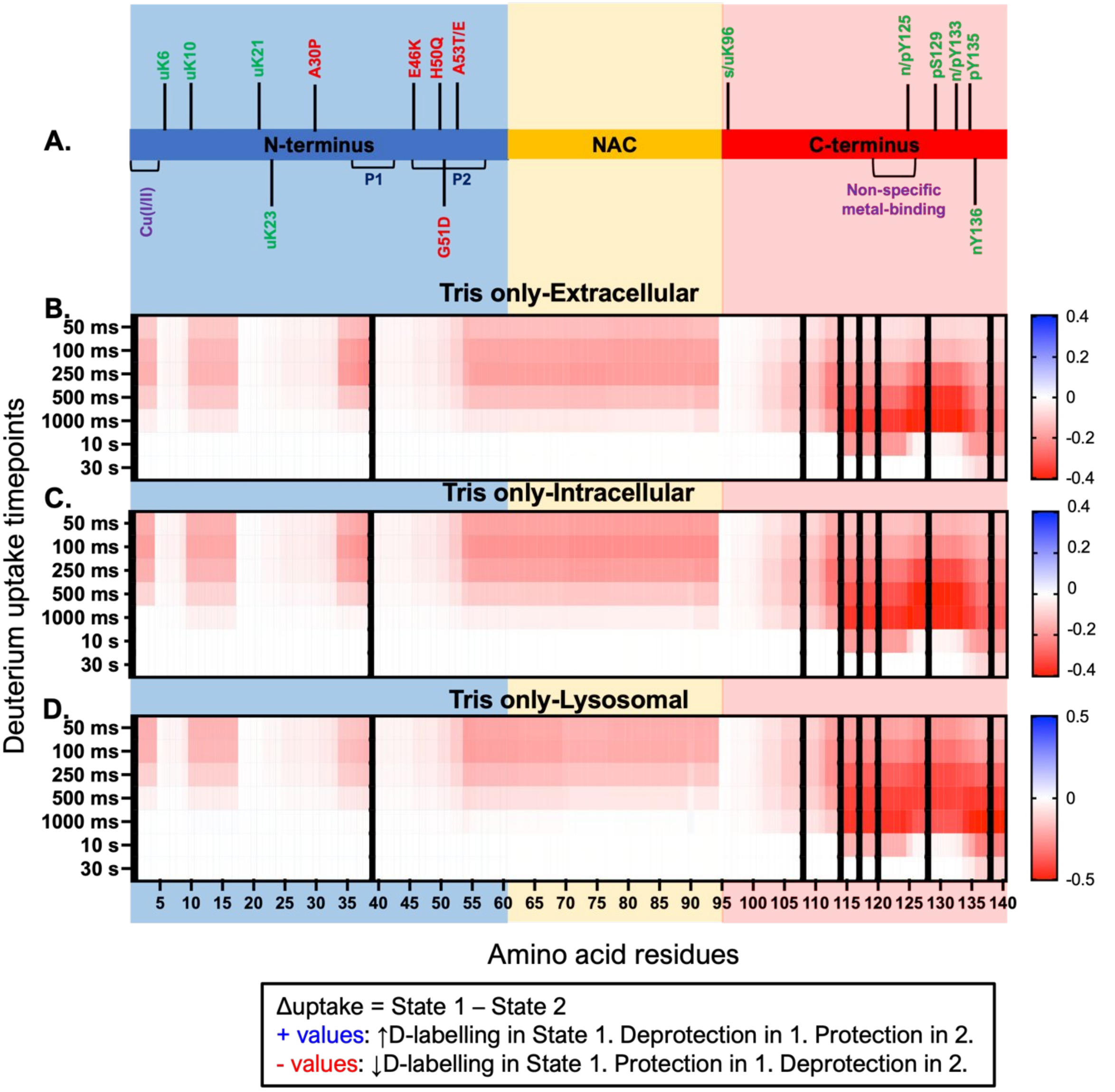

Figure S6: HDX-MS reveals localised differences in conformations of monomeric aSyn across Tris only vs the compartment conditions. (A) Schematic of aSyn monomer with important features and domains shown. (B-D) Heatmap showing significant differences in deuterium uptake per timepoint during an on-exchange reaction between STATE 1 – STATE 2 (title of each plot). Data are resolved to the amino acid level, down to single residues in certain regions. Positive values are in blue and represent increased uptake in STATE 1, whereas negative values are in red and represent increased uptake in STATE 2. Increased uptake indicates more solvent exposure and/or less participation in stable hydrogen-bonding networks. A possible explanation for the general protection across Tris-only could be that the salts in the cellular and extracellular buffers might be charge-shielding the N- and C-termini, such that in Tris-only, devoid of any biological salts, would adopt a more closed conformation due to increased intramolecular interactions.

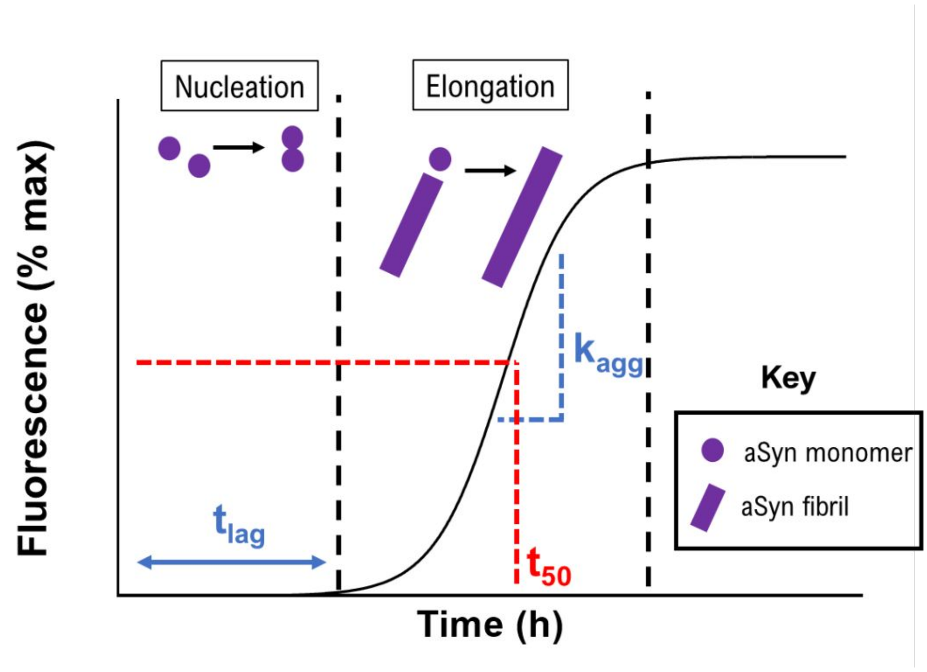

Figure S7: Schematic of the aggregation phases of nucleation (lag time) and elongation (slope of curve).

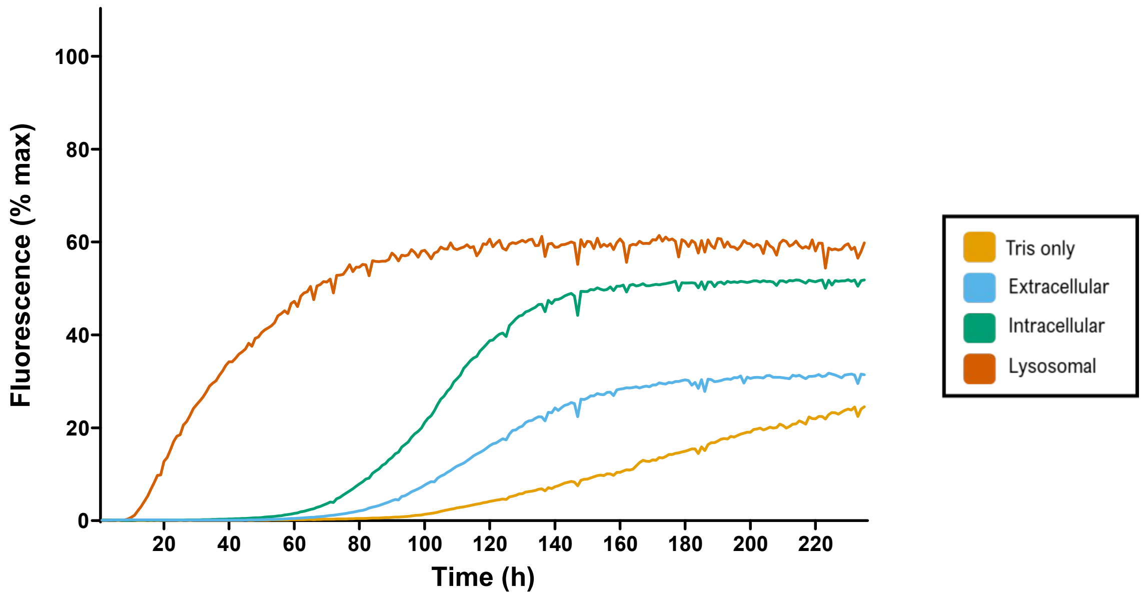

Figure S8: Average of three biological replicates upholds the same trend as summary data in Figure 1. Aggregation kinetics of aSyn in Tris only (yellow), extracellular (blue), intracellular (green) and lysosomal (orange) solution conditions were measured using ThT fluorescence intensity and plotted as % of maximum fluorescence at 480 nm. Trace shows the average of up to 9 technical replicates for three biological replicates (Summary data shown in Figure 1); A monomer concentration of 40 µM and ThT concentration of 40 µM were used. Measurements taken every hour, with 5 min shaking before the reading. Excitation/emission at 440/480 nm, gain settings 800, 1200, 1300, 1400, 1500. Assay length 235 h. Temperature set at 37 ˚C.

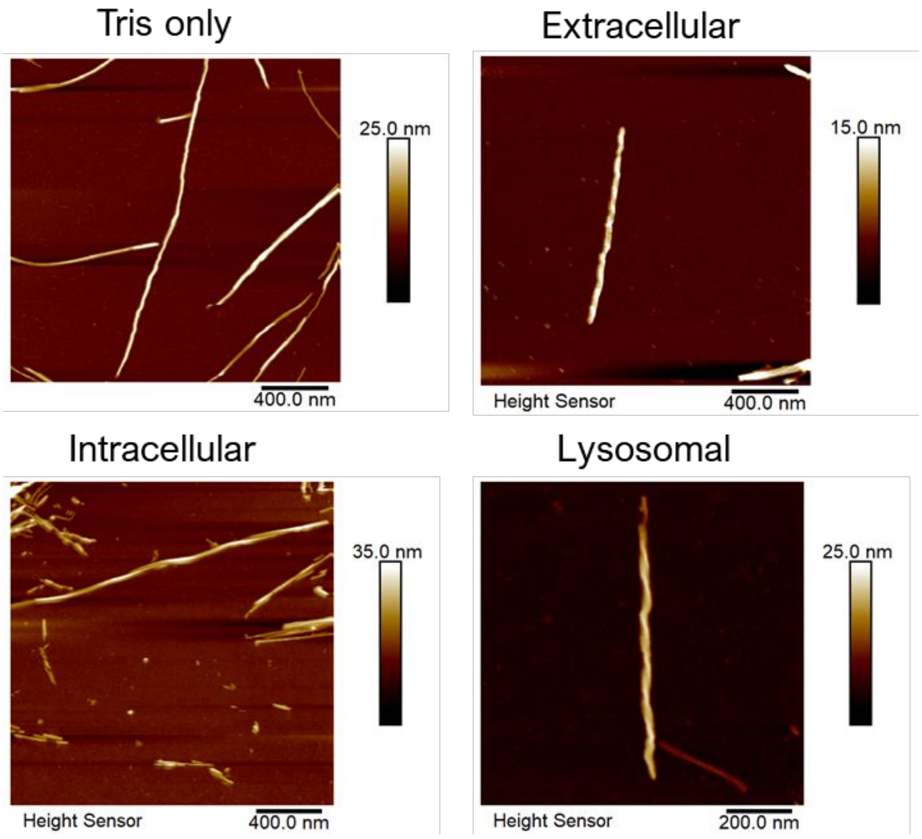

Figure S9: Representative AFM images of the main fibril polymorph in each condition. Note scale bars and height colour bars are not identical.

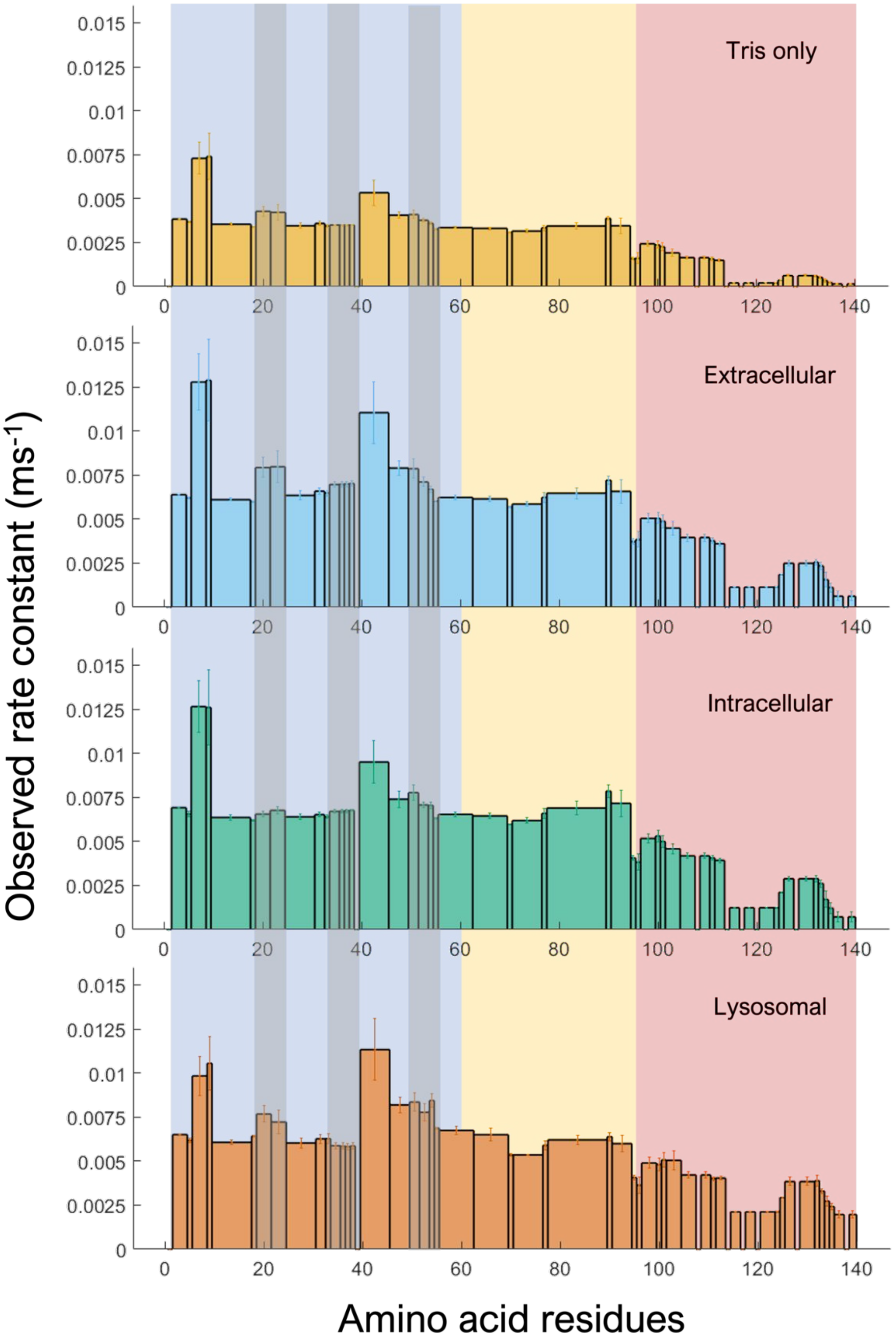

Figure S10: HDX-MS reveals different conformations in monomeric aSyn across all the conditions. Bars represent the observed hydrogen-exchange rate constant, k_{obs}, localised to the amino acids covered by the width of each bar. Error bars denote +/- 0.5 standard deviations. The N-terminus is shaded in blue, the NAC region in yellow and the C-terminus of aSyn in red. Data are for one technical replicate of three biological replicate aSyn samples. Grey boxes highlight example local features that are structurally dynamic, as evidenced by the alteration in the pattern of HDX observed rate constant between the conditions.

Table S1: Pearson correlation coefficients R along aSyn protein sequence (including ETD data) for correlation analyses between observed rate constant kobs from HDX-MS and nucleation lag time (t_{lag}) and elongation rate (k_{agg}) from ThT assays.

	Correlation coefficients			Correlation coefficients			Correlation coefficients			Correlation coefficients	
**Residue no.**	k_{agg}	t_{lag}	**Residue no.**	k_{agg}	t_{lag}	**Residue no.**	k_{agg}	t_{lag}	**Residue no.**	k_{agg}	t_{lag}
**1**	NaN	NaN	**36**	0.669	-0.643	**71**	0.710	-0.674	**106**	0.748	-0.846
**2**	0.840	-0.667	**37**	0.664	-0.636	**72**	0.710	-0.674	**107**	0.748	-0.846
**3**	0.840	-0.667	**38**	0.664	-0.634	**73**	0.710	-0.674	**108**	NaN	NaN
**4**	0.840	-0.667	**39**	NaN	NaN	**74**	0.710	-0.674	**109**	0.748	-0.846
**5**	0.846	-0.699	**40**	0.627	-0.840	**75**	0.710	-0.674	**110**	0.748	-0.846
**6**	0.688	-0.408	**41**	0.627	-0.840	**76**	0.710	-0.674	**111**	0.767	-0.832
**7**	0.688	-0.408	**42**	0.627	-0.840	**77**	0.763	-0.684	**112**	0.780	-0.853
**8**	0.688	-0.408	**43**	0.627	-0.840	**78**	0.776	-0.687	**113**	0.780	-0.853
**9**	0.747	-0.478	**44**	0.627	-0.840	**79**	0.776	-0.687	**114**	NaN	NaN
**10**	0.830	-0.728	**45**	0.627	-0.840	**80**	0.776	-0.687	**115**	0.574	-0.938
**11**	0.830	-0.728	**46**	0.716	-0.855	**81**	0.776	-0.687	**116**	0.574	-0.938
**12**	0.830	-0.728	**47**	0.716	-0.855	**82**	0.776	-0.687	**117**	NaN	NaN
**13**	0.830	-0.728	**48**	0.716	-0.855	**83**	0.776	-0.687	**118**	0.574	-0.938
**14**	0.830	-0.728	**49**	0.716	-0.855	**84**	0.776	-0.687	**119**	0.574	-0.938
**15**	0.830	-0.728	**50**	0.720	-0.857	**85**	0.776	-0.687	**120**	NaN	NaN
**16**	0.830	-0.728	**51**	0.720	-0.857	**86**	0.776	-0.687	**121**	0.574	-0.938
**17**	0.830	-0.728	**52**	0.747	-0.895	**87**	0.776	-0.687	**122**	0.574	-0.938
**18**	0.822	-0.805	**53**	0.747	-0.895	**88**	0.776	-0.687	**123**	0.574	-0.938
**19**	0.591	-0.756	**54**	0.732	-0.930	**89**	0.776	-0.687	**124**	0.582	-0.942
**20**	0.591	-0.756	**55**	0.795	-0.859	**90**	0.765	-0.548	**125**	0.670	-0.945
**21**	0.591	-0.756	**56**	0.800	-0.829	**91**	0.776	-0.600	**126**	0.672	-0.948
**22**	0.655	-0.726	**57**	0.800	-0.829	**92**	0.776	-0.600	**127**	0.672	-0.948
**23**	0.655	-0.726	**58**	0.800	-0.829	**93**	0.776	-0.600	**128**	NaN	NaN
**24**	0.655	-0.726	**59**	0.800	-0.829	**94**	0.776	-0.600	**129**	0.672	-0.948
**25**	0.767	-0.770	**60**	0.800	-0.829	**95**	0.773	-0.834	**130**	0.672	-0.948
**26**	0.767	-0.770	**61**	0.800	-0.829	**96**	0.645	-0.769	**131**	0.672	-0.948
**27**	0.767	-0.770	**62**	0.800	-0.829	**97**	0.634	-0.785	**132**	0.678	-0.947
**28**	0.767	-0.770	**63**	0.798	-0.805	**98**	0.634	-0.785	**133**	0.666	-0.937
**29**	0.767	-0.770	**64**	0.798	-0.805	**99**	0.634	-0.785	**134**	0.602	-0.947
**30**	0.767	-0.770	**65**	0.798	-0.805	**100**	0.678	-0.778	**135**	0.559	-0.928
**31**	0.771	-0.776	**66**	0.798	-0.805	**101**	0.700	-0.848	**136**	0.454	-0.872
**32**	0.771	-0.776	**67**	0.798	-0.805	**102**	0.703	-0.895	**137**	0.454	-0.872
**33**	0.766	-0.793	**68**	0.798	-0.805	**103**	0.703	-0.895	**138**	NaN	NaN
**34**	0.679	-0.653	**69**	0.798	-0.805	**104**	0.703	-0.895	**139**	0.454	-0.872
**35**	0.679	-0.653	**70**	0.731	-0.629	**105**	0.748	-0.846	**140**	0.454	-0.872

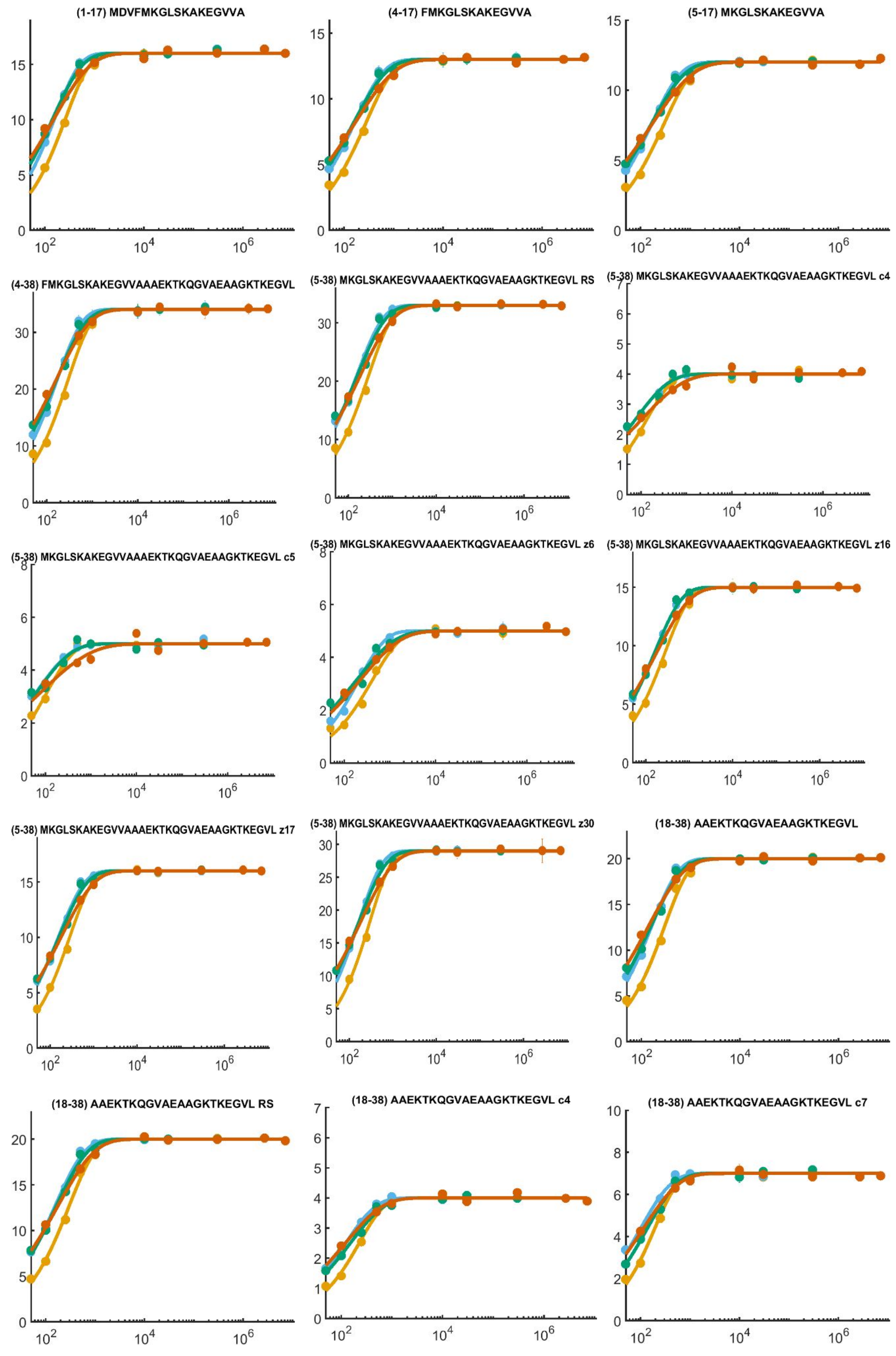
(1-17) MDVFMKGLSKAKEGVVA
(4-17) FMKGLSKAKEGVVA
(5-17) MKGLSKAKEGVVA
(4-38) FMKGLSKAKEGVVAAAEKTKQGVAEAAGKTKEGVL
(5-38) MKGLSKAKEGVVAAAEKTKQGVAEAAGKTKEGVL RS
(5-38) MKGLSKAKEGVVAAAEKTKQGVAEAAGKTKEGVL c4
(5-38) MKGLSKAKEGVVAAAEKTKQGVAEAAGKTKEGVL c5
(5-38) MKGLSKAKEGVVAAAEKTKQGVAEAAGKTKEGVL z6
(5-38) MKGLSKAKEGVVAAAEKTKQGVAEAAGKTKEGVL z16
(5-38) MKGLSKAKEGVVAAAEKTKQGVAEAAGKTKEGVL z17
(5-38) MKGLSKAKEGVVAAAEKTKQGVAEAAGKTKEGVL z30
(18-38) AAEKTKQGVAEAAGKTKEGVL
(18-38) AAEKTKQGVAEAAGKTKEGVL RS
(18-38) AAEKTKQGVAEAAGKTKEGVL c4
(18-38) AAEKTKQGVAEAAGKTKEGVL c7

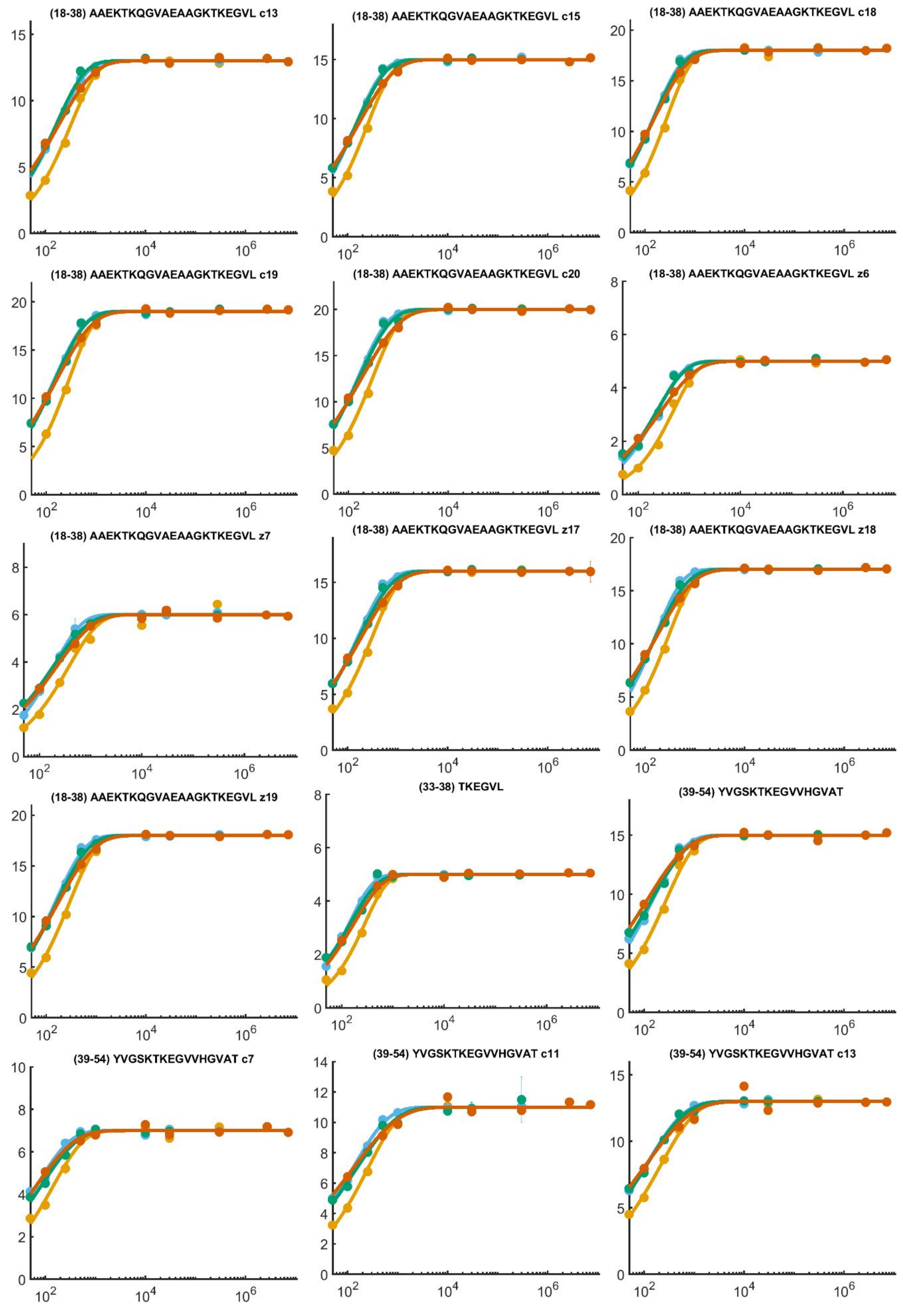
(18-38) AAEKTKQGVAEAAGKTKEGVL c13
(18-38) AAEKTKQGVAEAAGKTKEGVL c15
(18-38) AAEKTKQGVAEAAGKTKEGVL c18
(18-38) AAEKTKQGVAEAAGKTKEGVL c19
(18-38) AAEKTKQGVAEAAGKTKEGVL c20
(18-38) AAEKTKQGVAEAAGKTKEGVL z6
(18-38) AAEKTKQGVAEAAGKTKEGVL z7
(18-38) AAEKTKQGVAEAAGKTKEGVL z17
(18-38) AAEKTKQGVAEAAGKTKEGVL z18
(18-38) AAEKTKQGVAEAAGKTKEGVL z19
(33-38) TKEGVL
(39-54) YVGSKTKEGVVHGVAT
(39-54) YVGSKTKEGVVHGVAT c7
(39-54) YVGSKTKEGVVHGVAT c11
(39-54) YVGSKTKEGVVHGVAT c13

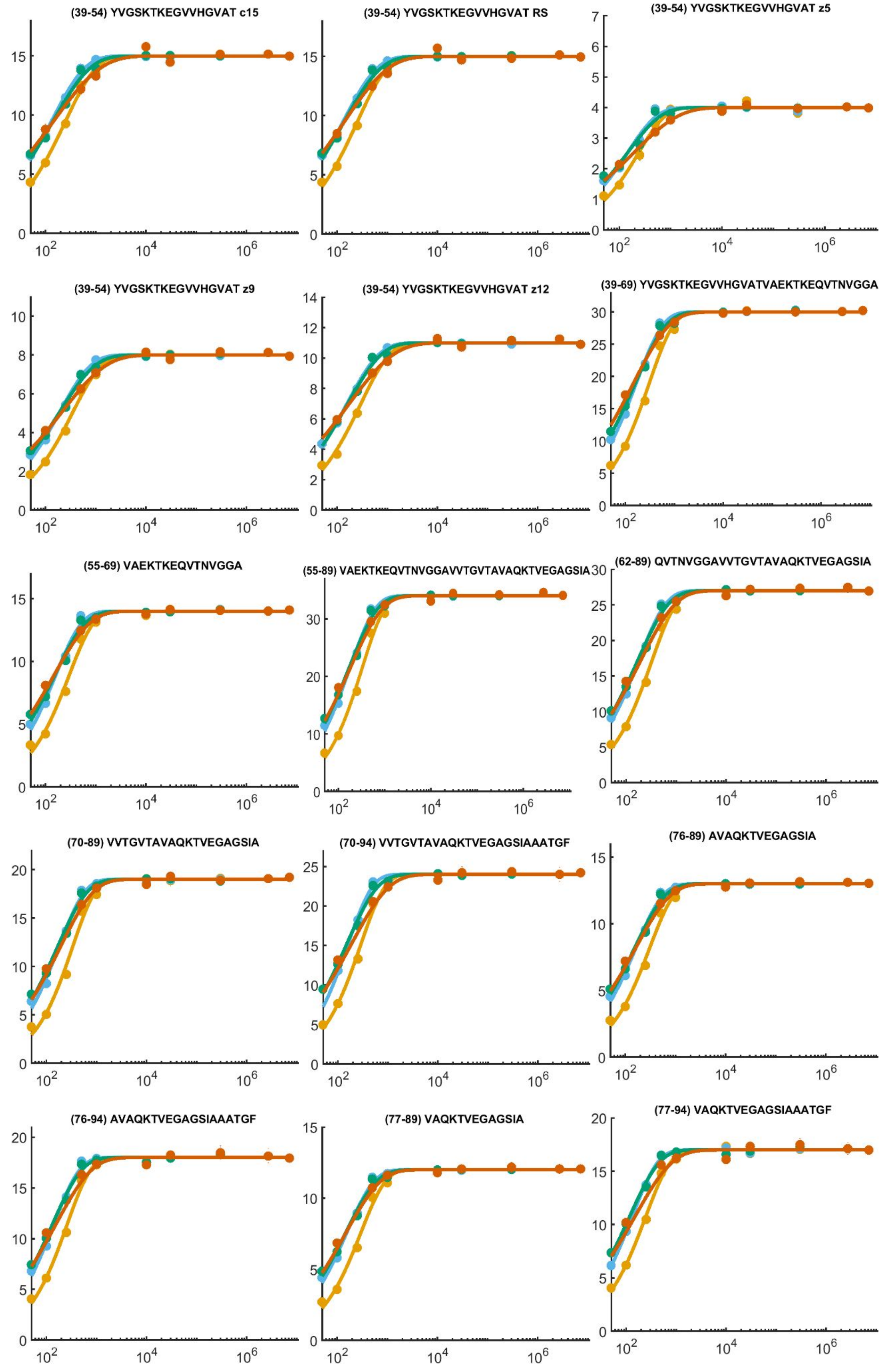
(39-54) YVGSKTKEGVVHGVAT c15
(39-54) YVGSKTKEGVVHGVAT RS
(39-54) YVGSKTKEGVVHGVAT z5
(39-54) YVGSKTKEGVVHGVAT z9
(39-54) YVGSKTKEGVVHGVAT z12
(39-69) YVGSKTKEGVVHGVATVAEKTKEQVTNVGGA
(55-69) VAEKTKEQVTNVGGA
(55-89) VAEKTKEQVTNVGGAVVTGVTAVAQKTVEGAGSIA
(62-89) QVTNVGGAVVTGVTAVAQKTVEGAGSIA
(70-89) VVTGVTAVAQKTVEGAGSIA
(70-94) VVTGVTAVAQKTVEGAGSIAAATGF
(76-89) AVAQKTVEGAGSIA
(76-94) AVAQKTVEGAGSIAAATGF
(77-89) VAQKTVEGAGSIA
(77-94) VAQKTVEGAGSIAAATGF

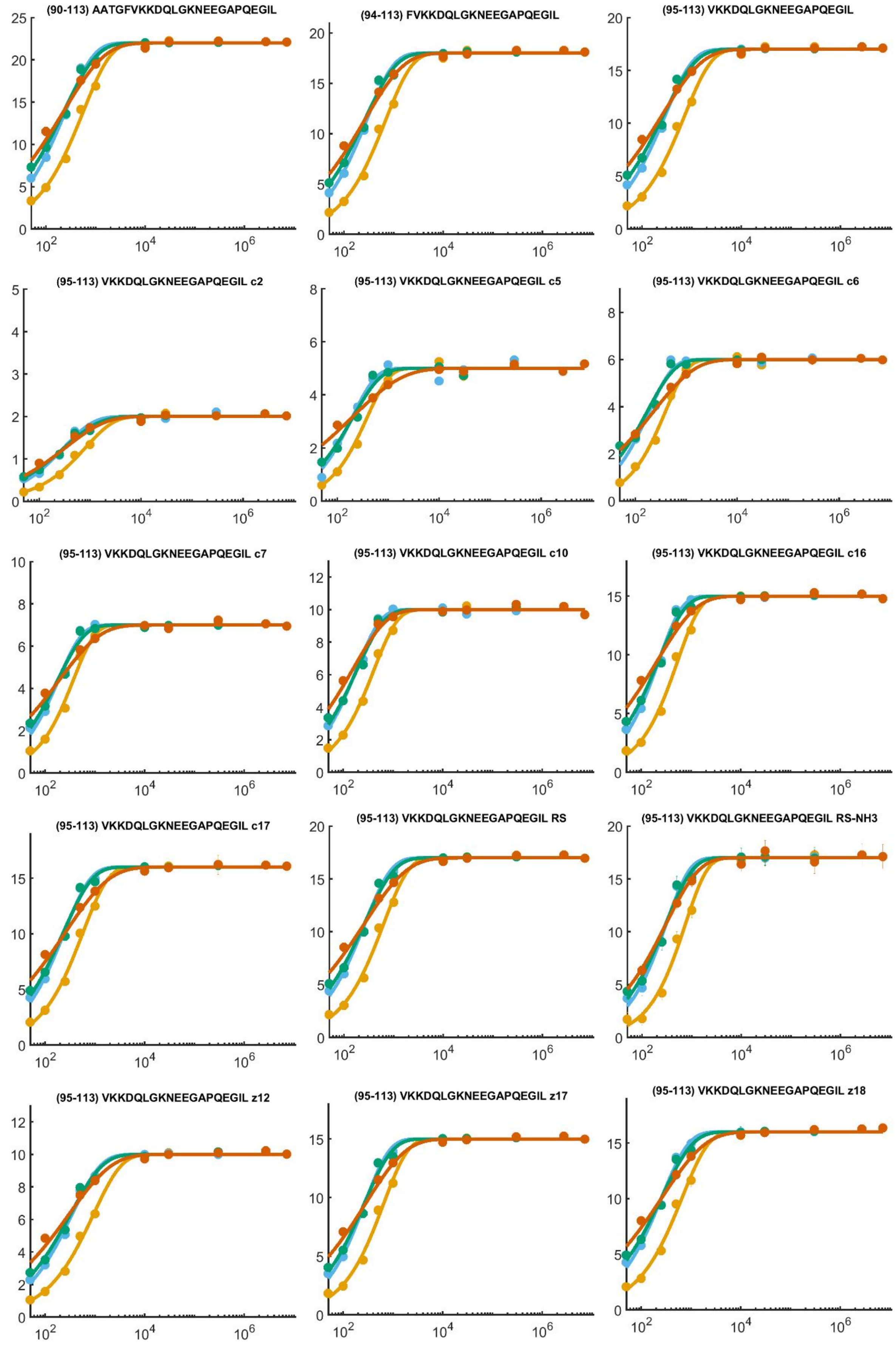
(90-113) AATGFVKKDQLGKNEEGAPQEGIL
(94-113) FVKKDQLGKNEEGAPQEGIL
(95-113) VKKDQLGKNEEGAPQEGIL
(95-113) VKKDQLGKNEEGAPQEGIL c2
(95-113) VKKDQLGKNEEGAPQEGIL c5
(95-113) VKKDQLGKNEEGAPQEGIL c6
(95-113) VKKDQLGKNEEGAPQEGIL c7
(95-113) VKKDQLGKNEEGAPQEGIL c10
(95-113) VKKDQLGKNEEGAPQEGIL c16
(95-113) VKKDQLGKNEEGAPQEGIL c17
(95-113) VKKDQLGKNEEGAPQEGIL RS
(95-113) VKKDQLGKNEEGAPQEGIL RS-NH3
(95-113) VKKDQLGKNEEGAPQEGIL z12
(95-113) VKKDQLGKNEEGAPQEGIL z17
(95-113) VKKDQLGKNEEGAPQEGIL z18

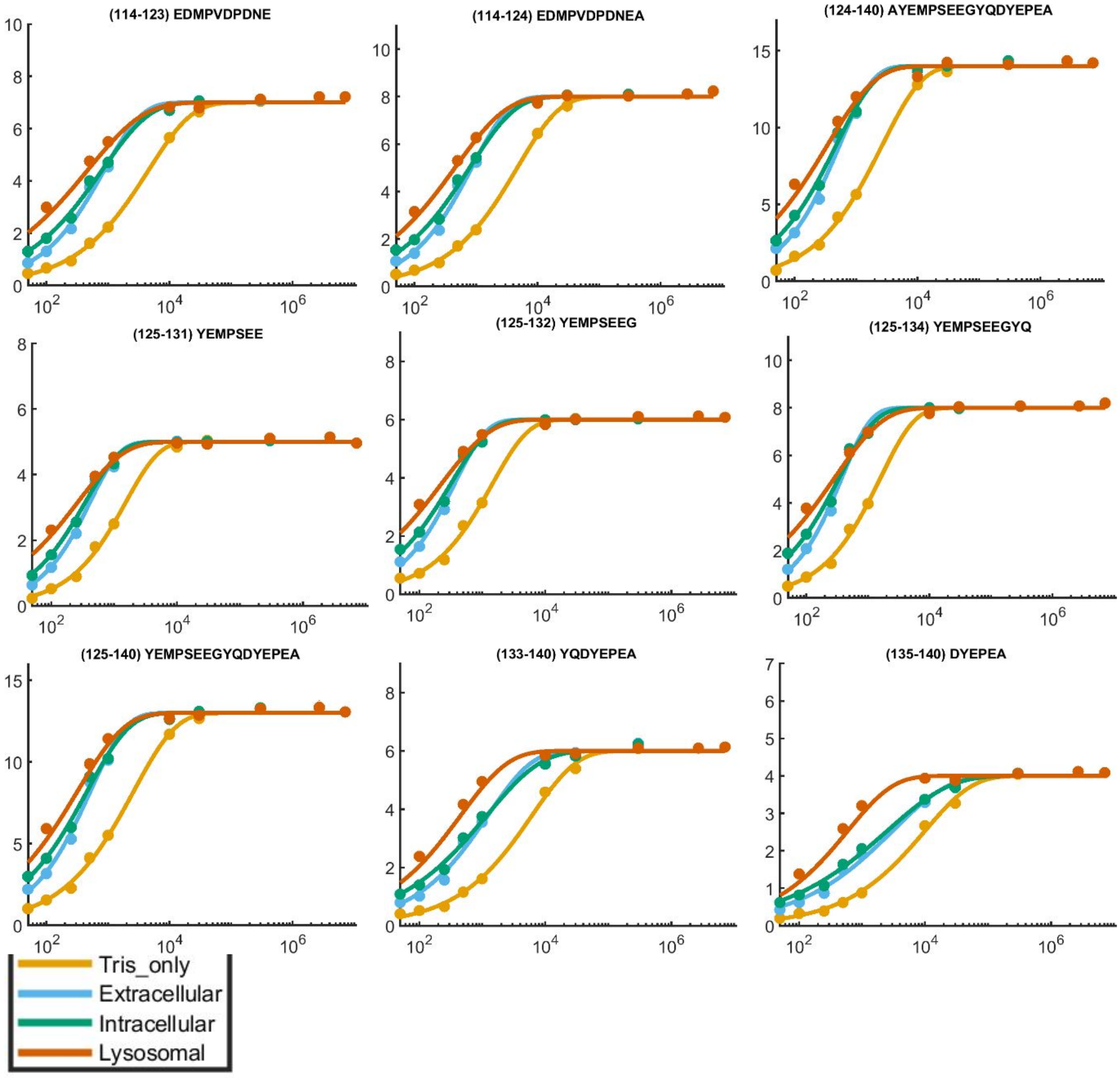

Figure S11: Empirically adjusted deuterium uptake plots for aSyn equilibrated in Tris only, extracellular, intracellular, and lysosomal buffer conditions. For Tris only, extracellular and intracellular states, timepoints collected were 50 ms, 100 ms, 250 ms, 500 ms, 1000 ms, 10 s, 30 s, 300 s. For lysosomal state, timepoints collected were 100 ms, 500 ms, 1000 ms, 10 s, 30 s, 300 s, 2700 s and 7200 s. Y-axis shows deuterium incorporation in Da and x-axis shows the log scale labelling times in ms (labels omitted for space). Data points are the mean of n=3 biological replicates (separate batches of aSyn protein).

Table S2: Number of fibril polymorphs (n) identified per condition.

	Tris only	Extracellular	Intracellular	Lysosomal
**Polymorph p1**	5	3	6	6
**Polymorph p2a**	8	-	11	-
**Polymorph p2b**	20	-	2	-
**Polymorph p3a**	-	12	2	16
**Polymorph p3b**	-	-	3	6
**Polymorph p4**	-	-	-	-

Table S3: HDX-MS experimental technical details.

Data Set	aSyn
HDX reaction details	See Table 1 for buffer conditions. 20˚C
HDX time course (ms)	Tris only, Extracellular, Intracellular: 50, 100, 250, 500, 1000, 10000, 30000, 300000.<br>Lysosomal: 100, 500, 1000, 10000, 30000, 300000, 2700000, 7200000.
HDX control samples	Maximal deuterium uptake plateau determination
Back-exchange (mean / IQR)	40.7% / 17.5%
# of Peptides	30
Sequence coverage	100%
Peptide Redundancy	3.79
Replicates (biological or technical)	3 (biological), 1(technical)
Significant differences in HDX (delta HDX > X Da)	0.36 (95% CI, quartiles method of outlier removal)

Code used to calculate the Pearson correlation coefficients (MATLAB 2021b, MathWorks, USA)

Calculate Pearson correlation coefficients between HDX k_{obs} and t_{lag} and k_{agg}

```
load kobs_vs_ThT_correlations_peptide_ETD.mat

%% Define colours
c = [0.4940 0.1840 0.5560; 0.6350 0.0780 0.1840; 0.9290 0.6940 0.1250;0 0.4470 0.7410];

%% Observed rate constant vs tlag
parfor a = 1:size(all_kobs,1)

    all_R_tlag{a,1} = corrcoef(all_kobs(a,:),all_tlag(a,:),'Alpha',0.01);
    all_r_tlag(a,1) = all_R_tlag{a,1}(2);

end

%% Observed rate constant vs slope
parfor a = 1:size(all_kobs,1)

    all_R_slope{a,1} = corrcoef(all_kobs(a,:),all_slope(a,:),'Alpha',0.01);
    all_r_slope(a,1) = all_R_slope{a,1}(2);
end
```

Draw correlation plots at each amino acid

```
fasta = fastaread('aSN_WT.fasta');
sequence = fasta.Sequence;

%% Define colours
c = [86/255 180/255 233/255; 0/255 158/255 115/255; 213/255 94/255 0 ; 230/255 159/255 0;
   86/255 180/255 233/255; 0/255 158/255 115/255; 213/255 94/255 0 ; 230/255 159/255 0;
   86/255 180/255 233/255; 0/255 158/255 115/255; 213/255 94/255 0 ; 230/255 159/255 0];

%% Observed rate constant vs tlag
parfor a = 1:size(all_kobs,1)

    if all_kobs(a,:) == 0
       continue
    end
    scatter(all_kobs(a,:),all_tlag(a,:),700,c,'filled');
    ax = gca;
    ax.YLabel.String =  'Lag time (h)'; % 'Slope (h^-1)';
    ax.XLabel.String = 'Observed rate constant (s^-1)';
    ax.LineWidth = 4;
    ax.FontSize = 30;
    %ax.XLim = [0 0.01];
    ax.YLim = [0 150];
    ax.XScale = 'linear';
    set(gcf,'Position',[493 206 1000 735]);

    % Add correlation line
    h1 = lsline(ax);
    h1.Color = 'k';
    h1.LineWidth = 5;
    h1.LineStyle = '--';
```

```
    title(sprintf('%s%d',fasta.Sequence(a),a));
    %legend([h(:)],{'Extracellular','Intracellular','Lysosomal','Tris only'});
    saveas(gcf,sprintf('kobs_vs_tlag_AA_%d.png',a));

end

%% Observed rate constant vs slope
parfor a = 1:size(all_kobs,1)

   if kobs(a,:) == 0
       continue
    end
    scatter(kobs(a,:),slope(a,:),700,c,'filled');
    ax = gca;
    ax.YLabel.String = 'Slope (h^-1)';
    ax.XLabel.String = 'Observed rate constant (s^-1)';
    ax.LineWidth = 4;
    ax.FontSize = 30;
    ax.YLim = [0.01 0.05];

    % Add correlation line
    h1 = lsline(ax);
    h1.Color = 'k';
    h1.LineWidth = 5;
    h1.LineStyle = '--';

    set(gcf,'Position',[493 206 1000 735]);
    title(sprintf('%s%d',fasta.Sequence(a),a));
    %legend([h(:)],{'Extracellular','Intracellular','Lysosomal','Tris only'});
    saveas(gcf,sprintf('kobs_vs_slope_AA_%d.png',a));

end
```

References

(1) Stephens, A. D.; Zacharopoulou, M.; Schierle, G. S. K. The Cellular Environment Affects Monomeric α-Synuclein Structure. *Trends Biochem. Sci.* **2018**, *44* (5), 453–466. https://doi.org/10.1016/j.tibs.2018.11.005 PMID - 30527975.

(2) Bonechi, C.; Ristori, S.; Martini, G.; Martini, S.; Rossi, C. Study of Bradykinin Conformation in the Presence of Model Membrane by Nuclear Magnetic Resonance and Molecular Modelling. *Biochim. Biophys. Acta - Biomembr.* **2009**, *1788* (3), 708–716. https://doi.org/10.1016/J.BBAMEM.2008.12.019.

(3) Kyle, D. J.; Martin, J. A.; Farmer, S. G.; Burch, R. M. Design and Conformational Analysis of Several Highly Potent Bradykinin Receptor Antagonists. *J. Med. Chem.* **1991**, *34* (3), 1230–1233. https://doi.org/10.1021/jm00107a052.
